# Tensile Property and Parametric Study of Heuristic Weaving Structures

**DOI:** 10.3390/s26196080

**Published:** 2026-09-25

**Authors:** Jing Xiao, Qiqi Li, Fang Wang, Tiefang Zou, Kai Gao, Lin Hu

**Affiliations:** 1 School of Transportation, Changsha University of Science & Technology, Changsha 410114, China; 2 College of Mechanical and Vehicle Engineering, Changsha University of Science & Technology, Changsha 410114, China

**Keywords:** heuristic weaving structures, blocking nets, ductile damage criterion, deformation pattern, tensile load

## Abstract

**Highlights:**

**What are the main findings?**

- Geometric parameters directly govern the structural load capacity and fracture modes.
- The 1.00 mm core increases peak tensile force by 9.6%, and hybrid SWR-S3 achieves 99.76% TFE.

**What are the implications of the main findings?**

- Parameter-driven structural tailoring effectively buffers localized stress peaks.
- The validated FEA framework provides a reliable predictive tool for structural optimization.

**Abstract:**

Heuristic weaving structures (HWSs) have attracted wide attention in engineering fields due to their high energy absorption capacity, reliable load-bearing performance, and highly adjustable deformation characteristics. This study systematically investigates the mechanical properties of HWSs under different boundary constraints and sparsity degrees via finite element analysis (FEA). The results indicate that all models maintain stable force transmission and reliable structural integrity throughout the loading process. Boundary constraints and sparsity significantly affect the mechanical properties and energy absorption of HWSs. Under different boundary constraints, the tensile specific energy absorption (TSEA) increases consistently from 8616.84 J/kg to 15,212.20 J/kg, representing a 76.54% improvement. Under varying sparsity degrees, the TSEA rises continuously from 8616.84 J/kg to 15,468.65 J/kg (a 79.52% increase), while the tensile energy absorption (TEA) also exhibits an enhancement from 22.83 J to 67.44 J (a 195.40% increase). For the out-of-plane load-bearing (X-axis) performance of HWSs, weakening boundary constraints promotes stress redistribution and cooperative deformation, thereby enhancing energy absorption and load-bearing efficiency. In summary, the proposed HWSs exhibit substantial bearing and energy absorption performance improvements. This work enriches the design methodology of woven energy-absorbing structures and provides theoretical guidelines for the parametric design and potential future application of HWSs in lightweight and energy-absorbing engineering applications.

## 1. Introduction

With highway traffic increasingly extending into mountainous and hilly regions, frequent accidents caused by out-of-control vehicles on long downhill slopes pose a serious threat to traffic safety and road order. As the ultimate safety facility for runaway vehicles, emergency escape ramps have received extensive attention regarding their protective performance [1,2,3]. As the core energy-absorbing component of cable-type escape ramps, blocking nets directly govern the stopping performance [4]. HWSs, as emerging structures with a bionic weaving design [5,6,7], exhibit both tunable deformation behavior and favorable mechanical properties [8,9]. Recently, the development of bio-inspired woven and architected mechanical metamaterials has demonstrated tremendous potential for customizable energy absorption. Advanced weaving-inspired structural designs leverage complex topological interlocking and tailored energy dissipation mechanisms to achieve exceptional damage tolerance and impact resistance [10]. Situating the proposed HWS within this broader metamaterial context highlights the distinct advantages of our plain weft-knitted configuration, particularly its capacity to engineer progressive strain pathways and maximize tensile specific energy absorption (TSEA) under constrained boundary conditions. This unique characteristic shows promising potential to satisfy the key demands of escape ramp blocking nets for energy absorption, flexible buffering, and lightweight design.

In recent years, advances in experimental and numerical simulation techniques, such as in situ impact tests and explicit dynamic simulations, have been achieved [11,12,13]. These developments have enabled systematic investigations into the dynamic response, damage mechanisms, and energy dissipation characteristics of metal woven meshes under impact loading [14,15,16]. Researchers have further examined individual factors influencing the mechanical behavior of such meshes, including impact location, impactor geometry, and structural parameters such as mesh size, wire diameter, and weaving pattern [17,18]. Correspondingly, various targeted numerical modeling approaches, performance evaluation frameworks, and damage control strategies have been proposed [19,20,21,22]. Collectively, these efforts have established a solid technical foundation for the engineering design and practical deployment of metal woven meshes in flexible protective systems.

The weaving structure critically governs the arresting net’s mechanical behavior. Common forms include diamond mesh, which offers the best impact resistance and exhibits funnel-shaped deformation under static indentation [23]. Hexagonal twisted mesh adopts a double-twisted structure and can withstand rockfall even when individual steel wires are broken [24]. Ring net is composed of interconnected steel wire rings, providing excellent energy dissipation for rockfall protection [25]. Multi-layer composite mesh adopts a double-layer design with a buffer rubber layer at least 15 mm thick, significantly enhancing impact absorption [26]. Energy absorption mainly comes from wire deformation due to internal energy and sliding friction at cross points. Impact location, clamping orientation, and projectile size significantly affect efficiency [27]. An entangled wire mesh damper consisting of a cap-shaped damper in series with a parallel spring effectively improves the impact reliability of engineering equipment [28]. For steel wire ring nets, a membrane equivalent method using a virtual constitutive model reproduces the macroscopic mechanical responses under rockfall impact, providing an efficient analytical tool for designing large-scale flexible protection systems [29]. For fiber-reinforced metal mesh laminates under low-velocity impact, an effective analysis model has been developed by combining the Johnson–Cook damage model and a surface contact-based cohesive zone method [30]. Among these representative contributions, Zhu et al. [31] developed a discrete element model to simulate the response of flexible net systems. Coulibaly [32] observed that overall mesh barrier stiffness and residual energy dissipation capacity increase with successive impacts. Zhang [33] proposed a residual performance evaluation method that accounts for cumulative plastic deformation and local wire damage.

Parallel to these material-level investigations, extensive research has been conducted on the failure modes, influencing factors, and performance optimization of flexible escape lane blocking nets incorporating heuristic woven structures [34,35,36,37,38]. Yu Zhixiang et al. [39] conducted a field investigation along a mountainous highway in western China. They identified five typical failure modes. These include steel column buckling, connection failure between the mesh and cables, mesh damage, steel cable fracture, and anchor point failure. Mentani et al. [40] highlighted that the dynamic response of such systems is highly dependent on vehicle impact conditions, with variations in vehicle load, impact speed, and impact position significantly affecting energy transfer efficiency and damage evolution. Luo et al. [41] emphasized that improper connections between the woven mesh and adjacent components restrict structural deformation capacity and reduce overall energy absorption, constituting a primary cause of system failure. Research on out-of-control vehicle impacts has further demonstrated that vehicle shape and size critically influence both vehicle motion during impact and the mechanical response of the blocking net [42,43,44]. Impact tests with varying vehicle types and angles reveal that vehicles with sharp or irregular front profiles tend to induce stress concentrations, leading to localized mesh damage and increased penetration risk [45,46]. Notably, a counterintuitive “bullet effect” has been observed, wherein smaller vehicles with reduced overall impact energy paradoxically exhibit a higher probability of penetrating the net due to the concentration of impact force [47]. In terms of performance assessment, Lambert et al. [48] developed a meta model-based prediction framework for evaluating interception capability. Marcelli et al. [49] proposed a multi-level damage assessment scheme incorporating aging, corrosion, and repeated impact effects.

While extensive research has been conducted on metal woven meshes using various analytical methods and performance evaluation schemes [50,51,52,53,54,55], current methodologies are constrained by their focus on isolated variables such as impact position or block shape. This renders them incapable of capturing the synergistic effects of multi-factor coupled loading that characterize actual out-of-control vehicle or rockfall impacts. More importantly, the fundamental question of how structural parameters influence the cascade from localized material damage to overall system failure remains unresolved. Addressing this critical gap, this study introduces an HWS inspired by plain weft-knitted configurations. This novel structure is characterized by its enhanced tensile toughness and a distinctive stable stepwise deformation behavior, which offers inherent advantages for energy absorption under complex loading. This research systematically investigates the effects of key structural parameters on tensile properties. The aim is to clarify the relationship between structural configuration and mechanical performance. This work provides a theoretical basis for moving beyond empirical design. It supports the shift toward rational, performance-based parametric study of flexible protective systems.

This study is organized as follows. Section 2 shows the geometry and inspiration source of the HWSs. The FEA and experimental results are presented in Section 3. Section 4 discusses the effects of key parameters, including boundary constraint conditions, porosity, and loading direction, on the mechanical properties and deformation modes of HWSs. Section 5 gives the conclusions of this study.

## 2. Establishment of Geometric Model

This section elaborates on the modeling process of the HWS. Figure 1a illustrates the design inspiration for the HWS. The structural design of the HWS draws primary inspiration from the plain weft-knitted structure in weft-knitted configurations: it employs the interlaced morphology of plain weft-knitting as its basic unit, merging the attributes of lattice structures and woven structures. It thus inherits both the lightweight and high-strength advantages of lattice structures and the superior energy absorption performance and mass production compatibility of woven structures. This aligns with the plain weft-knitted structure depicted in Figure 1a. The modular woven architecture of the HWS confers key engineering benefits. It can directly leverage the mature production logic of everyday plain weft-knitted fabrics. It also enables unit-based modeling via the simulated plain weft-knitting structure. This streamlines subsequent analysis and parametric study of mechanical properties. Figure 1b illustrates the modeling process of the modular bionic lattice structure. Sketches are created on the YZ plane and XZ plane. These sketches are then extruded along their normal direction. This operation yields the cell curves. These cell curves are taken as guide curves. A cell is generated by sweeping a circular profile. The cells are arrayed in the width and height directions. Adjacent cells on the outer side are then connected with arcs. The structural parameters of the basic HWS model are shown in Table 1, where *W* represents the width of a single unit, *H* represents its height, T represents its thickness, h1 denotes the lateral spacing between units, Z indicates the number of horizontal rows in the steel wire mesh, Y represents the number of vertical columns in the mesh, and d refers to the diameter of the individual wire strands.

Furthermore, HWSs have broad potential application prospects. As shown in Figure 1c, HWSs can be deployed to mountain roads, bridges and expressways, serving as protective and blocking structures to safeguard passing vehicles.

## 3. Simulation Analysis and Tensile Experiments

In this section, the mechanical characteristics of a single steel wire rope and the HWS are studied.

### 3.1. The Mechanical Properties of Steel Wire Rope

To characterize the mechanical properties of a single wire rope, three identical tensile samples were prepared with a length of 300 mm and a diameter of 0.48 mm. Tensile tests were performed using a CMT-5105GL universal testing machine (Hunan Xiangyu Measurement and Control Technology Co., Ltd., Changsha, China) at a loading rate of 2 mm/min under room temperature conditions, and axial clip-on electronic extensometer (Model YYU-10/50, NCS, China, gauge length = 50 mm) was adopted to measure the axial deformation of steel wire rope. Figure 2a shows the test setup and single wire samples, and Figure 2b presents the samples after tensile testing. The resulting stress–strain curves are displayed in Figure 2c.

Based on the incompressibility assumption (i.e., constant specimen volume during stretching), the Young’s modulus of the single wire was calculated using the following equation:

(1) $$E=\frac{\sigma_{T}}{\varepsilon_{T}}=\frac{\sigma_{e}(1+\varepsilon_{e})}{\ln(1+\varepsilon_{e})}=\frac{F_{0}(1+\Delta l_{1}/l_{1})}{\ln(1+\Delta l_{1}/l_{1})h_{1}w},$$

where $F_{0}$ is the tensile force, $\Delta l_{1}$ is the elongation of the sample’s middle length, and *ε_e_*, σ*_e_*, *ε_T_* and $\;\sigma_{T}\;$ represent the engineering strain, engineering stress, true strain, and true stress in the length direction, respectively. The density of the wire rope is 7.85 × 10^3^ kg/m^3^, and according to Equation (1), the Young’s modulus of the wire rope can be calculated as 174,968 MPa.

The material parameter definition requires specification of both the initial damage criterion and damage evolution law. To accurately simulate the damage and fracture behavior of steel wire ropes under tensile overload, this study employs the ductile damage criterion [56] with corresponding evolution settings. The ductile criterion represents a phenomenological model that predicts damage initiation resulting from void nucleation, growth, and coalescence within the material matrix. The model formulates the equivalent plastic strain at damage initiation (${\bar{\dot{\varepsilon }}}^{pl}$) as a function of stress triaxiality (η) and strain rate ($\dot{\varepsilon }$), as expressed in Equation (2). Damage initiation occurs when the condition in Equation (3) is satisfied, with subsequent evolution calculated incrementally according to Equation (4).

(2) $${\bar{\varepsilon }}_{D}^{pl}\left(\eta ,{\bar{\dot{\varepsilon }}}^{pl}\right),$$

(3) $$\omega_{D}=\int \frac{d{\bar{\varepsilon }}^{pl}}{{\bar{\varepsilon }}_{D}^{pl}\left(\eta ,{\bar{\dot{\varepsilon }}}^{pl}\right)}=1,$$

(4) $$\omega_{D}=\frac{\Delta {\bar{\varepsilon }}^{pl}}{{\bar{\varepsilon }}_{D}^{pl}\left(\eta ,{\bar{\dot{\varepsilon }}}^{pl}\right)}\geq 0,$$

where $\eta =-\frac{p}{q}\;$ is the stress triaxiality, *p* is the pressure, *q* is the von Mises equivalent stress, ${\bar{\dot{\varepsilon }}}^{pl}$ is the equivalent plastic strain rate, and *w*_D_ is a state variable that increases monotonically with plastic deformation.

The fracture simulation of ductile metals requires three critical parameters for damage initiation: η, Lode parameter ($\bar{\theta }$) and the fracture strain ($\varepsilon_{f}$). These parameters are determined through the following constitutive equations:

(5) $$\eta =\frac{\sigma_{m}}{\sigma_{eq}}=\frac{\sigma_{1}+\sigma_{2}+\sigma_{3}}{3\sigma_{eq}},$$

(6) $$\bar{\theta }=\frac{2\sigma_{2}-\sigma_{1}-\sigma_{3}}{\sigma_{1}-\sigma_{3}},$$

(7) $$\varepsilon_{f}=\ln\left(\frac{1}{1-RA}\right),$$

where $\sigma_{m}$ represents the hydrostatic stress, ${\sigma_{m}=(\sigma }_{1}+\sigma_{2}+\sigma_{3})/3$, and $\sigma_{eq}$ denotes the von Mises equivalent stress $\sigma_{eq}=\sqrt{\frac{1}{2}\left[{\left(\sigma_{1}-\sigma_{2}\right)}^{2}+{\left(\sigma_{2}-\sigma_{3}\right)}^{2}+{\left(\sigma_{3}-\sigma_{1}\right)}^{2}\right]}$, with $\sigma_{1}$, $\sigma_{2}$, and $\sigma_{3}$ being the principal stresses. RA indicates the reduction in area. Under uniaxial tension conditions, the stress triaxiality $\eta =\frac{1}{3}$ and the normalized Lode angle parameter $\bar{\theta }=1$. The fracture strain $\varepsilon_{f}$ calculated via Equation (7) yields a value of 0.85.

Damage evolution in this context involves the removal of elements upon reaching predefined failure criteria, either in terms of critical displacement or fracture energy, thereby simulating material separation. For the current simulation, the critical failure displacement was specified as 0.1 mm to achieve realistic fracture propagation. It should be noted that the Johnson–Cook plasticity parameters (A, B, n) and the damage evolution settings were systematically calibrated by fitting the true stress–strain data obtained from the single-wire tensile experiments. The high fidelity of these selected parameters is subsequently validated by the excellent agreement between the experimental and FEA force–deformation curves in Section 3.3. Table 2 shows the finalized material parameters of the steel wire rope.

### 3.2. The Setup of FEA and Experiment

The finite element models of the single steel wire rope are presented in Figure 3a. The specimen model was constructed with a length of 50 mm (matching the 50 mm extensometer used in experiments) and meshed using 0.1 mm hexahedral elements (C3D8R). The single steel wire model comprised 15,984 elements, and numerical simulations were conducted using ABAQUS/Explicit 2022 (Dassault Systèmes, Vélizy-Villacoublay, France). Boundary conditions matched the experimental setup. One end was fully constrained, and the other end was subject to axial displacement. History output variables and field output variables are configured to output the load and displacement borne by the single steel wire rope. In the simulation, the ratio of kinetic energy to internal energy was kept below 5% to ensure that the solution is valid.

### 3.3. The Results of FEA and Experiments

Key energy absorption metrics analyzed in this study include tensile energy absorption (TEA), tensile specific energy absorption (TSEA), peak tensile force (PTF), mean tensile force (MTF), and tensile force efficiency (TFE). Their mathematical definitions are as follows:

(8) $$TEA=\int_{0}^{s}F\left(x\right)dx,$$

(9) $$TSEA=\frac{TEA}{SM},$$

(10) $$MTF=\frac{TEA}{s},$$

(11) PTF=max(F(t)),

(12) $$TFE=\frac{MTF}{PTF}\times 100\%,$$

where s is the maximum deformation, and $F\left(x\right)$ is the tensile force. TSEA is the ratio of TEA to structural mass (*SM*), and MTF is the ratio of TEA to s. To comprehensively evaluate the protective performance of HWSs, it is essential to understand the physical relationships among these metrics. TEA quantifies the absolute total energy dissipated during deformation, while TSEA normalizes this value by the *SM*, serving as a critical indicator for lightweight design efficiency. PTF represents the maximum load transmitted to the boundary supports; an excessively high PTF may cause premature failure of the anchoring system. MTF reflects the average resistance provided by the structure. Consequently, TFE acts as a crucial bridge between MTF and PTF to evaluate load-bearing stability. A high TFE indicates that the structure maintains a steady resistance close to its peak capacity without severe force fluctuations, which translates to smooth and reliable energy absorption.

Figure 3b presents the strain cloud diagram of a single wire rope under tensile loading. During initial loading, at 0.6 mm displacement, the strain distribution remains uniform with minimal plastic strain, consistent with elastic deformation following Hooke’s law. As displacement increases to 1.2–1.8 mm, macroscopic plastic strain begins to accumulate and localize, evident from the yellow–red color gradients in the contour plot, while maintaining overall macroscopic uniformity. At 2.4 mm displacement approaching fracture, the single wire rope exhibits classical ductile failure progression. This is characterized by pronounced macroscopic necking, where cross-sectional area reduction causes stress intensification, followed by stable plastic deformation until fracture. This is followed by stable plastic deformation until fracture. This sequence fully captures the predictable elastic–plastic–necking–fracture transition of homogeneous materials.

Figure 3c demonstrates that both the experiments and FEA exhibit consistent force–deformation curves for the single wire, characterized by an initial rise to peak load followed by progressive softening. This mechanical response indicates that the structures initially bear gradually increasing tensile loads until reaching maximum capacity, after which their load-bearing capability declines due to structural deformation and eventual failure. Specifically, the single wire approaches peak load at approximately 0.5–1.0 mm displacement. The TEA–deformation curves in Figure 3d reveal that during deformation from 0 mm to 2.5 mm, the energy absorption values increase progressively, with excellent agreement between experimental and FEA results. This close correlation validates the FEA model’s capability to accurately simulate the structures’ energy absorption mechanisms.

Table 3 presents a statistical comparison between experimental measurement and FEA. For the single wire rope, TEA, TSEA and PTF show excellent agreement with experimental results, and their deviations are −0.46%, −0.46% and −2.27%, respectively. Collectively, Figure 3 and Table 3 confirm that the FEA methodology reliably simulates the mechanical performance and energy absorption characteristics of the single wire rope. It should be noted that while these single-wire tests successfully validate the fundamental material constitutive and damage models, they do not encompass the complex inter-wire contact, friction, and macroscopic failure modes of the complete woven mesh.

### 3.4. FEA of HWS

The FEA model of the HWS is shown in Figure 4a. The HWS model is mounted on a frame via connecting rings. Simulation analysis is performed using ABAQUS/Explicit software. To ensure rigorous numerical reproducibility, specific boundary and loading conditions were applied. The fixed frame is set with a fully fixed constraint, meaning that all six translational and rotational degrees of freedom are rigidly restricted ($U_{x}=U_{y}=U_{z}=UR_{x}=UR_{y}=UR_{z}=0$). For the loading condition, a displacement-controlled load is applied to the reference point at the geometric center of the rigid hammer. The hammer moves smoothly along the negative X-axis direction at a predefined quasi-static constant velocity to act on the HWSs, while its remaining five degrees of freedom are constrained to prevent any unintended lateral deflection or rotation during the impact process. The HWSs use the monofilament material parameters obtained in Section 3.1. All other parts are set as rigid bodies. The meshing of the HWS model is shown in Figure 4b. The steel wire mesh part adopts hexahedral elements (C3D8R) with a mesh size of 0.1 mm. To ensure numerical stability and accuracy, a mesh sensitivity analysis was conducted on the single-wire model using global element sizes of 0.05 mm, 0.1 mm, 0.15 mm, and 0.2 mm (the detailed mesh sensitivity analysis is provided in Figure S1 of the Supplementary Materials). Comparing the simulated mechanical responses with the experimental single-wire tensile data revealed that the 0.1 mm mesh size provided the optimal balance, demonstrating excellent agreement with the experimental force–deformation curves while maintaining high computational efficiency. Finer meshes (0.05 mm) yielded negligible accuracy gains at a severe computational cost, while coarser meshes (0.15 mm and 0.2 mm) prematurely underestimated the peak tensile force. Therefore, a consistent element size of 0.1 mm was applied to all macroscopic HWS configurations in this parametric evaluation. The remaining parts use tetrahedral elements (C3D10M). The mesh size of the hammer is set to 0.5 mm, that of the connecting rings to 0.17 mm, and that of the fixed frame to 0.2 mm. The overall model is set as general contact. The tangential friction behavior is modeled using the penalty formulation, with a contact friction coefficient of 0.115. This specific value is not arbitrary; it was adopted based on established experimental tribological measurements for smooth-finish, cold-drawn steel wires [57], accurately reflecting the unlubricated, bright surface condition of the experimental specimens. Maintaining this constant, experimentally validated baseline friction coefficient isolates the structural variables, allowing for a pure evaluation of topological sensitivities without confounding contact non-linearities. Furthermore, because the slender individual wires predominantly experience uniaxial tension, the ductile damage initiation is calibrated using a single fracture strain derived directly from the uniaxial tensile tests (the detailed damage parameter sensitivity analysis is provided in Figure S2 of the Supplementary Materials), effectively capturing the dominant failure mode under this specific high-triaxiality stress state.

To guarantee the validity of the quasi-static assumption within the explicit dynamic solver, the energy balance was rigorously monitored for all macroscopic HWS simulations. Throughout the entire loading history of every configuration, the ratio of kinetic energy to internal energy was strictly maintained below 5%, confirming that dynamic inertial effects remained negligible and the quasi-static response was accurately captured.

Figure 4c shows the stress distribution contour plot of the HWS model. As can be seen from the stress distribution contour plot, the stress at the contact part between the hammer and the steel wire mesh is the largest. As the hammer moves along the negative X-axis direction, the contact area between the hammer and the steel wire mesh gradually increases. The deformation of the steel wire mesh tends to spread outward with the contact point as the center. The steel wire mesh gradually encloses the hammer at 14 mm. The stress distribution shifts from the contact point to the contact surface. As can be seen from the color changes in the contour plot, during the loading process from 0 mm to 14 mm, the parts around the hammer are subjected to relatively large stress; after 14 mm, the steel wire mesh connected to the connecting rings also bears large stress. Finally, partial fracture occurs in the steel wire mesh around the contact point at 28 mm. The fracture occurs because the stress on the steel wire mesh exceeds its load limit. Figure 4d shows the load–displacement curve of the HWS model. It can be seen from the curve that the load on the steel wire mesh reaches a peak within the displacement range of 0–5 mm, with a subsequent downward trend. After 5 mm, the load rises steadily until it reaches the peak of the load–displacement curve. The point corresponding to this peak also marks the initial fracture moment of the steel wire mesh. After that, as the hammer continues to stretch, the load on the steel wire mesh drops rapidly until the hammer breaks through and separates from the steel wire mesh, at which point the load drops to 0 N.

Table 4 shows the numerical predictions derived from the finite element axial tensile simulations for HWSs. Because the full HWS response involves complex contact, friction, and large topological deformation, these macroscopic structural results are presented strictly as numerical predictions based on the validated single-wire material model. HWSs have excellent energy absorption characteristics, with relatively high TSEA values, and the advantages of lightweight design are significant.

## 4. Parameter Analysis of HWSs

In this section, the effects of key parameters, namely boundary constraint condition, sparsity degree, and loading direction, on the mechanical properties and deformation modes of HWSs are explored. While this study focuses on the internal parametric study of HWSs, it is important to contextualize these mechanical responses against established woven architectures. Conventional configurations, such as diamond meshes, typically exhibit funnel-shaped deformation under out-of-plane loading, whereas ring nets rely on the interconnected sliding of steel wire rings for energy dissipation. In contrast, the plain weft-knitted topology of the HWS introduces a distinct stepwise deformation behavior. The following parametric analysis primarily evaluates how internal geometric adjustments optimize this specific bionic topology, establishing a foundational dataset for future direct benchmarking against conventional diamond, hexagonal, and ring net architectures under equivalent mass and impact conditions.

### 4.1. The Out-of-Plane Load-Bearing Properties of HWSs Under Coupled Structural Scaling

Sparsity degrees are set for four configurations. The core layout of the overall structure remains unchanged. For simplicity and clarity of reference, these HWS configurations with different sparsity degrees are designated as HWS, HWS-S1, HWS-S2, and HWS-S3. Figure 5 depicts the sketches of their front views. Detailed dimensional parameters are presented in Table 5. In these configurations (HWS to HWS-S3), a coupled structural scaling strategy is employed. Rather than isolating spatial sparsity, multiple interconnected variables are modified simultaneously: the characteristic geometric dimensions (W, H) and the wire diameter (d) are progressively increased, while the total lattice count (Z×Y) is proportionally reduced to fit the fixed macroscopic frame. Therefore, the mechanical responses observed in these models are not solely attributable to lattice sparsity, but rather to the synergistic effects of a thicker wire diameter, enlarged unit cells, and reduced geometric constraint density.

Table 6 and Figure 6 present the FEA results for HWS, HWS-S1, HWS-S2, and HWS-S3. As we progress from HWS to HWS-S3, coordinated changes in key performance metrics are observed: TEA, TSEA, MTF, and PTF all exhibit continuous growth, while TFE shows moderate fluctuations across the series. TEA shows a steady and significant increase, rising from 22.83 J for HWS to 67.44 J for HWS-S3. This monotonic growth demonstrates a consistent enhancement in the total energy absorption capacity as the structural design evolves from HWS to HWS-S3. Figure 6d further illustrates that HWS-S3 accumulates energy at a faster rate and reaches a higher final value, indicating superior energy absorption potential under progressive deformation. TSEA also increases consistently throughout the series, from 8616.84 J/kg for HWS to 15,468.65 J/kg for HWS-S3. This indicates that the optimized structure of HWS-S3 allows for more efficient energy utilization per unit mass, presenting a critical advantage for lightweight engineering applications. MTF increases monotonically from 878.25 N for HWS to 2189.72 N for HWS-S3, while PTF follows a parallel upward trend, rising from 1800.07 N to 5280.42 N. These trends confirm a substantial enhancement in the load-bearing capacity of the structures as the design progresses from HWS to HWS-S3.

The tensile force– and TEA–deformation curves for these HWS-S models are shown in Figure 6c,d. Forces for HWS, HWS-S1, HWS-S2, and HWS-S3 exhibit mechanical behavior dependent on the degree of coupled structural scaling. Notably, HWS shows fluctuating force responses in the early deformation stage. This indicates insufficient structural stability. In contrast, HWS-S3 displays a steeply ascending force curve with the highest peak. This reflects enhanced stiffness and load bearing capacity. This behavior is primarily governed by the coupled structural scaling: the smaller wire diameter and dense lattice in HWS lead to unstable initial load transfer, while the substantially thicker wire (d = 0.84 mm) combined with the sparser layout in HWS-S3 provides higher intrinsic stiffness, enabling coordinated structural deformation and sustained load enhancement. In terms of energy absorption, curves for all models increase monotonically with deformation, evidencing continuous energy absorption capacity. Notably, as the structural scaling degree increases from HWS to HWS-S3, the energy absorption growth rate consistently increases, with HWS-S3 having the steepest TEA–deformation slope. This indicates that configurations with a higher degree of structural scaling (e.g., thicker wire and sparser lattice) achieve faster energy absorption. This aligns with the progressive strain propagation and extensive plastic deformation observed in Figure 6e. It validates the structural benefits of this coupled scaling strategy in optimizing energy absorption efficiency.

Figure 6e,f present the deformation patterns of HWS, HWS-S1, HWS-S2, and HWS-S3. They reveal how the coupled structural scaling governs strain evolution through the coupling of structure, load, and deformation behavior. The fundamental mechanism involves structural elements initiating deformation under tensile loading. Strain propagates across the network through inter-element contact stresses. This represents a universal sequence characterized by progressive initiation and sparsity dependence. Crucially, the degree of structural scaling modulates this process. Configurations with a lower degree of structural scaling (e.g., smaller wire diameter and denser lattice) such as HWS exhibit weak geometric constraints. This enables relatively localized strain distribution and early failure-related strain concentration, as evidenced by the rapid development of high-strain regions in the early deformation stages at 7 mm and 14 mm and premature failure behavior. In contrast, configurations with a higher degree of structural scaling (e.g., thicker wire and sparser lattice) such as HWS-S3 demonstrate strong geometric constraints. These constraints promote stepwise strain propagation, creating delayed but extensive plastic strain distribution across the structural network, with strain gradually spreading from the load-bearing core to the surrounding elements as deformation progresses. This sparsity-dependent behavior follows a clear mechanistic chain. The sparsity degree directly affects the strength of geometric constraints within the structure. This further influences the efficiency of strain transfer between elements. It ultimately determines the macroscopic strain distribution and failure mode. Decreasing sparsity such as HWS promotes localized strain patterns with early failure characteristics, limiting the overall energy absorption capacity. In contrast, increasing sparsity such as HWS-S3 enhances strain synchronization and progressive deformation, enabling more uniform and extensive plastic straining. However, this comes at the cost of potential stress concentration risks in the early deformation stages due to the higher initial stiffness of the denser, more constrained structure. These strain evolution characteristics ultimately align with the force–deformation and TEA–deformation responses observed in Figure 6c,d. The localized strain and early failure in HWS correspond to its lower peak force and energy absorption capacity. HWS-S3 exhibits progressive and extensive strain propagation. This is associated with its higher peak force, sustained load-bearing capacity, and enhanced energy absorption efficiency. This conclusively demonstrates how the sparsity degree structurally engineers the strain pathways within the HWS network, directly tailoring the macroscopic performance in terms of load capacity and energy absorption.

### 4.2. The Out-of-Plane Load-Bearing Properties of HWSs Under Various Boundary Constraints

To investigate the effect of the number of connecting rings on the structural boundary constraints of HWSs, this section presents four configuration schemes with different numbers of connecting rings. Configuration 1 is as follows: 10 connecting rings are arranged at the left transverse end, 9 connecting rings at the right transverse end, and 5 connecting rings at each of the upper and lower longitudinal ends. In configuration 2, the number of connecting rings at the upper and lower longitudinal ends is reduced to half of the baseline value. In configuration 3, the number of connecting rings at the left and right transverse ends is reduced to half of the baseline value. In configuration 4, the number of connecting rings at both the upper and lower longitudinal ends and the left and right transverse ends is reduced to half of the baseline value. The core structure of the steel wire woven mesh remains unchanged. For simplicity and clarity of reference, these HWS configurations are named HWS, HWS-X1, HWS-X2 and HWS-X3. Figure 7 depicts the front view of these HWS models. Figure 7 illustrates that altering the number of connecting rings directly modifies the structural boundary constraints.

Table 7 and Figure 8 present the FEA results for HWS, HWS-X1, HWS-X2 and HWS-X3. TEA exhibits a monotonic increase, rising from 22.83 J for HWS to 40.31 J for HWS-X3, indicating a substantial enhancement in the overall energy absorption capacity. Correspondingly, TSEA increases consistently from 8616.84 J/kg to 15,212.20 J/kg, demonstrating a remarkable improvement in mass-specific energy absorption efficiency. This improvement is attributed to the optimized structural design of the later configurations, which facilitates more effective plastic deformation. MTF also increases monotonically from 878.25 N for HWS to 1110.53 N for HWS-X3, confirming enhanced load-bearing capacity. While PTF shows slight variation, peaking at 1820.44 N for HWS-X1 and then decreasing to 1710.15 N for HWS-X3, this does not detract from the overall performance improvements. TFE rises from 48.79% to 64.94%, validating a more optimized energy utilization efficiency in the advanced configurations.

The tensile force– and TEA–deformation curves for these HWS-X models are shown in Figure 8c,d. Forces for HWS, HWS-X1, HWS-X2, and HWS-X3 demonstrate configuration-dependent mechanical behavior. Notably, HWS shows an early peaking force curve followed by a decrease. This indicates insufficient load bearing durability. In contrast, HWS-X1, HWS-X2, and HWS-X3 exhibit continuously rising force curves. Among them, HWS-X3 has the steepest slope. This reflects higher stiffness and better sustained load capacity. This behavior is primarily governed by the structural design of each HWS. Specifically, the HWS structure lacks the robustness for prolonged load bearing, while configurations from HWS-X1 to HWS-X3 feature optimized architectures that enable progressive stiffness and load enhancement. In terms of energy absorption, all models’ TEA–deformation curves increase monotonically with deformation, evidencing robust energy absorption capacity. Notably, as the configuration advances from HWS to HWS-X3, the growth rate of energy absorption consistently increases. HWS-X3’s TEA–deformation curve has the highest slope, indicating the fastest energy absorption rate, which correlates with its superior TEA 40.31 J and TSEA 15,212.20 J/kg performance in the previous analysis. This shows that the evolutionary design from HWS to HWS-X3 improves load-bearing capability. It also optimizes energy absorption efficiency through structural refinement. Ultimately, it provides a more effective and efficient energy absorbing system.

Figure 8e,f show the effective plastic strain contours and deformation patterns of HWS, HWS-X1, HWS-X2, and HWS-X3. HWS displays a relatively localized strain distribution. At failure, its plastic strain concentrates in specific regions. This indicates insufficient structural synergy and inefficient load transfer. In contrast, HWS-X1, HWS-X2, and HWS-X3 demonstrate more extensive and stepwise strain propagation, with plastic deformation gradually spreading across the structure as deformation increases. Notably, HWS-X3 shows the most intensive and well-coordinated strain evolution, with plastic strain spreading across numerous structural elements at failure, reflecting a highly synergistic deformation mechanism. HWS’s structure results in localized strain patterns with weak coordination, leading to premature stress concentration and limited energy dissipation. As the configuration progresses to HWS-X1, HWS-X2, and HWS-X3, the boundary conditions have been adjusted. This adjustment improves strain synchronization and propagation efficiency, enabling more uniform stress distribution and progressive plastic deformation. This enhanced strain coordination aligns with the superior force–deformation and energy absorption performances observed in Figure 8c,d, where HWS-X3 exhibits the highest load capacity and energy absorption efficiency. Reducing the number of surrounding connecting rings lowers the boundary constraint intensity. Stresses originally concentrated at the constrained ends are redistributed along the weaving paths, engaging more wire strands in cooperative load transfer. The weaker constraint also promotes greater geometric rearrangement of mesh openings and inter-strand slippage, thereby delaying localized necking and fracture. As a result, total energy absorption, peak tensile force, and tensile fracture efficiency increase substantially. Thus, for HWS, stronger constraints do not inherently improve performance. Instead, appropriately reducing boundary constraints exploits the structure’s large-deformation flexibility and progressive stiffening, leading to higher energy absorption efficiency. The evolution from HWS to HWS-X3 therefore represents a rational design that engineers favorable strain pathways, ensures uniform and coordinated plastic deformation, and achieves tailored macroscopic improvements in load capacity and energy absorption.

### 4.3. The Y-Axis Tensile Properties of HWSs Under Different Boundary Constraints

For these four different configurations, the core structure of the steel wire woven array remains unchanged. For ease of understanding, we denote these HWS models with different boundary constraints as HWS-Y, HWS-X1-Y, HWS-X2-Y, and HWS-X3-Y. In these Y-axis tensile simulations, a bilateral uniform displacement-controlled loading condition is applied. Specifically, the connecting rings located at the top and bottom longitudinal ends are simultaneously subjected to equal and opposite predefined displacements along the Y-axis to stretch the structure symmetrically. Figure 9 shows the front views of these HWS models, where the boundary constraint ranges under different configurations are indicated by distinct markings, such as the orange dashed boxes. It can be seen from Figure 9 that variations in boundary constraints directly affect the constraint range of the structure, thereby significantly influencing its tensile properties in the Y-axis.

Table 8 and Figure 10 present the FEA results for HWS-Y, HWS-X1-Y, HWS-X2-Y, and HWS-X3-Y. When evaluating the series from HWS-Y to HWS-X3-Y, distinct performance changes are observed. TEA, TSEA, and MTF show a general decreasing trend after an initial peak. PTF declines in parallel with them. TFE displays a fluctuating pattern. TEA peaks at 10.73 J for HWS-Y, then decreases to 7.12 J for HWS-X1-Y, rises slightly to 9.60 J for HWS-X2-Y, and finally drops to 6.83 J for HWS-X3-Y. This indicates that HWS-Y has the strongest total energy absorption capacity, with subsequent configurations showing reduced performance, except for moderate recovery in HWS-X2-Y. TSEA shows maximum mass-specific energy absorption of 4047.40 J/kg for HWS-Y. It maintains a significant advantage over HWS-X1-Y (2688.04 J/kg), HWS-X2-Y (3621.13 J/kg), and HWS-X3-Y (2576.98 J/kg). This is attributable to HWS-Y’s higher mass-to-stiffness ratio, enabling increased energy absorption per unit mass. MTF decreases from 746.17 N for HWS-Y to 472.40 N for HWS-X1-Y, then rises to 622.31 N for HWS-X2-Y, and finally drops to 410.10 N for HWS-X3-Y. This reflects a non-monotonic reduction in maximum load-bearing capacity as the configuration moves from HWS-Y to HWS-X3-Y. PTF also follows a similar non-monotonic trend, from 1312.37 N for HWS-Y to 680.68 N for HWS-X1-Y, rising to 960.49 N for HWS-X2-Y, and declining to 624.58 N for HWS-X3-Y. This further validates the configuration-dependent load-bearing characteristics, with HWS-Y exhibiting the highest peak load resistance. TFE is lowest for HWS-Y at 58.23%, then increases to 69.04% for HWS-X1-Y, decreases slightly to 64.79% for HWS-X2-Y, and rises marginally to 65.66% for HWS-X3-Y. This indicates that while HWS-Y has the highest total energy absorption, it exhibits lower force efficiency compared to other configurations, suggesting a tradeoff between total absorption and efficiency.

The tensile force– and TEA–deformation curves for these HWS-Y models are shown in Figure 10c,d. Forces for HWS-Y, HWS-X1-Y, HWS-X2-Y, and HWS-X3-Y exhibit configuration-dependent mechanical behavior. Notably, HWS-Y shows a higher initial force peak and subsequent fluctuations. This indicates its distinct initial stiffness and structural stability. In contrast, HWS-X1-Y, HWS-X2-Y, and HWS-X3-Y display relatively smooth force curves with progressive increases. These reflect their different load-bearing characteristics. This behavior is primarily governed by the structural design of each HWS: HWS-Y’s structure leads to early stiffness and unstable load transfer, while HWS-X1-Y, HWS-X2-Y, and HWS-X3-Y feature more coordinated deformation for sustained load enhancement. In terms of energy absorption, curves for all models increase monotonically with deformation, evidencing continuous energy absorption capacity. Notably, HWS-Y has the steepest TEA–deformation slope, indicating the fastest energy absorption rate, which aligns with its peak TEA performance of 10.73 J in the previous analysis. As the configuration changes from HWS-Y to HWS-X3-Y, the energy absorption growth rate generally decreases, with HWS-X3-Y showing the shallowest slope, reflecting reduced energy absorption efficiency in later configurations.

Figure 10e presents the deformation patterns of HWS-Y, HWS-X1-Y, HWS-X2-Y, and HWS-X3-Y. They reveal how configuration governs strain evolution through structure–load–deformation coupling. Physically, the boundary configurations directly dictate the allowable geometric rearrangement of the mesh. In tightly constrained models like HWS-Y, the restricted mobility of the wire strands prevents efficient load redistribution, forcing premature plastic strain concentration at localized defect points. In contrast, the relaxed boundaries in HWS-X3-Y allow the inter-woven elements to physically slide and reorient along the tensile axis. This kinematic freedom delays localized necking and engages a significantly larger volume of the structural network in cooperative energy dissipation. HWS-Y exhibits a relatively active and heterogeneous strain distribution. It shows obvious plastic strain concentration in later deformation stages at 28 mm and 35 mm. This indicates weak structural constraint and direct, unregulated strain propagation. In contrast, HWS-X1-Y, HWS-X2-Y, and HWS-X3-Y demonstrate a more coordinated and uniform strain distribution. HWS-X3-Y shows the most uniform strain propagation across structural elements during the entire deformation process. This reflects stronger geometric constraints and more synchronized element deformation. This configuration-dependent behavior follows a mechanistic chain: configuration affects constraint strength, which in turn influences strain transfer efficiency, and finally determines macroscopic strain distribution. HWS-Y’s weak constraint promotes localized strain patterns with early-stage concentration risks, which can lead to premature localized failure. As the configuration progresses to HWS-X1-Y, HWS-X2-Y, and HWS-X3-Y, enhanced constraint facilitates strain synchronization, delaying the onset of severe strain localization. However, this comes with the tradeoff of potentially reduced initial stiffness and peak load capacity, as observed in the force–deformation responses. These strain evolution characteristics ultimately align with the force–deformation responses in Figure 10c and energy absorption performance in Table 8 and Figure 10a. HWS-Y’s localized strain provides higher initial energy absorption and peak force. However, this comes at the cost of less uniform deformation. Conversely, HWS-X1-Y, HWS-X2-Y, and HWS-X3-Y achieve more sustained deformation through uniform strain propagation. However, they exhibit lower peak performance.

### 4.4. The Z-Axis Tensile Properties of HWSs Under Different Boundary Constraints

In this investigation of Z-axis tensile behavior, the core mesh structure is kept identical across all four models. To facilitate clear comparison, the configurations with varying boundary constraints are designated as HWS-Z, HWS-X1-Z, HWS-X2-Z, and HWS-X3-Z. For the Z-axis tensile evaluations, a bilateral uniform displacement-controlled loading condition is employed. The connecting rings positioned at the left and right transverse ends are simultaneously subjected to equal and opposite predefined displacements along the Z-axis to achieve symmetric in-plane stretching. Figure 11 shows the front views of these HWS models, where the boundary constraint ranges under different configurations are indicated by distinct markings, such as the orange dashed boxes. It can be seen from Figure 11 that variations in boundary constraints directly affect the constraint range of the structure, thereby significantly influencing its tensile properties in the Z-axis.

Table 9 and Figure 12 present the FEA results for HWS-Z, HWS-X1-Z, HWS-X2-Z, and HWS-X3-Z. Evaluation of the series from HWS-Z to HWS-X3-Z shows coordinated performance changes. TEA, TSEA, and MTF reach a peak at HWS-X1-Z. PTF displays a similar trend. TFE exhibits a fluctuating trend and achieves its highest value at HWS-Z. TEA shows a peak response at HWS-X1-Z, rising from 22.37 J for HWS-Z to 29.26 J for HWS-X1-Z, before decreasing to 14.58 J for HWS-X2-Z and slightly increasing to 15.41 J for HWS-X3-Z. This demonstrates enhanced total energy absorption capacity for HWS-X1-Z. TFE is highest for HWS-Z at 80.67%, then decreases to 74.60% for HWS-X1-Z, rises to 76.05% for HWS-X2-Z, and drops to 72.27% for HWS-X3-Z. This indicates that HWS-Z achieves the most optimized energy utilization efficiency, converting a larger proportion of input energy into useful absorption, while HWS-X1-Z prioritizes absolute energy absorption and load capacity over efficiency. These provide clear guidelines for application-specific design tradeoffs. HWS-X1-Z is preferred for scenarios prioritizing high energy absorption and load bearing. HWS-Z is suitable for applications valuing energy utilization efficiency. HWS-X2-Z and HWS-X3-Z offer a balance of controlled deformation and moderate performance.

The tensile force– and TEA–deformation curves for these HWS-Z models are shown in Figure 12c,d. Forces for HWS-Z, HWS-X1-Z, HWS-X2-Z, and HWS-X3-Z exhibit configuration-dependent mechanical behavior. Notably, HWS-X1-Z shows a steeply ascending force curve with a high peak. This reflects enhanced stiffness and load-bearing capacity. In contrast, HWS-Z displays early-stage fluctuations in force. This indicates differences in initial stiffness and structural stability during the initial deformation phase. HWS-X2-Z and HWS-X3-Z present relatively smooth force responses with lower peak forces, demonstrating distinct load-bearing characteristics characterized by more gradual force development and reduced peak loading. This behavior is primarily governed by the structural design of each HWS configuration. HWS-X1-Z’s optimized configuration enables coordinated load transfer and enhanced stiffness. This leads to a rapid rise in force and a high peak. HWS-Z features unstable early-stage load propagation. This results in force fluctuations. HWS-X2-Z and HWS-X3-Z adopt more gradual deformation mechanisms. These mechanisms distribute loads more evenly. They lead to smoother force responses. In terms of energy absorption, TEA–deformation curves for all models increase monotonically with deformation, evidencing continuous energy absorption capacity throughout the loading process. Notably, HWS-X1-Z has the steepest TEA–deformation slope, indicating the fastest energy absorption rate, which aligns with its peak TEA performance of 29.26 J in the previous analysis. As the configuration changes from HWS-Z to HWS-X3-Z, the energy absorption growth rate is most prominent in HWS-X1-Z, reflecting its optimized structural design for enhanced energy absorption efficiency. HWS-Z follows with a moderate energy absorption rate, while HWS-X2-Z and HWS-X3-Z exhibit the slowest energy accumulation, consistent with their lower TEA values of 14.58 J and 15.41 J, respectively. This trend underscores the critical role of structural configuration in dictating both the rate and total capacity of energy absorption.

Figure 12e presents the deformation patterns of HWS-Z, HWS-X1-Z, HWS-X2-Z, and HWS-X3-Z. They reveal how configuration governs strain evolution through structure–load–deformation coupling. From a physical perspective, the transition in boundary constraints fundamentally alters the internal stress transfer pathways. HWS-Z suffers from severe strain localization because heavy constraints lock the boundaries, causing sharp stress gradients. Conversely, the tailored constraints in HWS-X1-Z facilitate a more uniform internal load transfer mechanism. By permitting necessary geometric flexibility, the contact stresses at the weaving nodes are distributed more evenly, translating to the delayed onset of macroscopic failure and a higher capacity for sustained load-bearing. HWS-Z exhibits a relatively concentrated strain distribution. It shows significant plastic strain localization in later deformation stages, especially at the structural edges. This indicates weak structural constraint and direct strain propagation. These characteristics lead to early-stage strain concentration risks. In contrast, HWS-X1-Z, HWS-X2-Z, and HWS-X3-Z demonstrate more coordinated strain distribution. HWS-X1-Z shows distinct staged deformation. It exhibits uniform strain propagation across structural elements during mid-to-late deformation at 21 mm and 28 mm. This reflects stronger geometric constraints that promote synchronized strain transfer. HWS-X2-Z and HWS-X3-Z further exhibit gradual, evenly distributed strain evolution, with reduced localization compared to HWS-Z, indicating enhanced constraint strength as configuration evolves. This configuration-dependent behavior follows a mechanistic chain: configuration affects constraint strength, which in turn influences strain transfer efficiency, and finally determines macroscopic strain distribution. HWS-Z’s weak constraint promotes localized strain patterns with early-stage concentration risks, while HWS-X1-Z’s optimized configuration facilitates uniform strain propagation. As the configuration progresses to HWS-X2-Z and HWS-X3-Z, enhanced constraint facilitates strain synchronization, though this comes with the tradeoff of more gradual strain development relative to HWS-X1-Z. These strain evolution characteristics ultimately align with the force–deformation responses in Figure 12c. For example, HWS-X1-Z’s uniform strain propagation correlates with its steeper force rise and higher peak force, as coordinated strain transfer enables efficient load distribution across the structure. In contrast, HWS-Z’s localized strain leads to early force fluctuations and lower peak force, reflecting inefficient strain transfer and load distribution. This conclusively demonstrates how configuration structurally engineers strain pathways. It tailors macroscopic performance in energy absorption and load capacity. HWS-X1-Z’s strain coordination enables superior energy absorption and load bearing. HWS-Z’s strain localization limits its performance despite moderate efficiency.

### 4.5. Unified Anisotropic Deformation Mechanism

The disparate responses of HWSs under different loading directions highlight a unified anisotropic deformation mechanism governed by the plain weft-knitted topology. Under out-of-plane (X-axis) loading, the structure primarily absorbs energy through macroscopic membrane deflection. Weakening the boundary constraints facilitates inward material flow and geometric reorientation, which mitigates central stress concentration and significantly enhances total energy absorption. Conversely, under in-plane (Y-axis and Z-axis) loading, the structure relies on direct tensional load transfer at the boundaries. For longitudinal (Y-axis) tension, the loops immediately tighten, demanding strong, uniformly distributed boundary constraints (HWS-Y) to prevent premature edge failure. For transverse (Z-axis) tension, the loops must first uncurl and widen; a slight relaxation of the boundaries (HWS-X1-Z) provides the optimal kinematic freedom for this structural reorientation, achieving an intermediate performance peak before excessive constraint reduction triggers catastrophic stress concentration at the remaining connection points.

## 5. Conclusions

This study proposes a series of HWSs. The reliability of the fundamental material model was validated via single-wire tensile tests. Building upon this calibrated material data, FEA was utilized to numerically evaluate the effective load-bearing and energy absorption performance of the full HWS configurations. This study further systematically investigated the effects of boundary constraints and sparsity degrees on HWS tensile properties and revealed the underlying regulatory mechanisms. The main conclusions are summarized as follows:

1. Coupled structural scaling (the simultaneous adjustment of wire diameter, unit dimensions, and lattice density) significantly influences energy absorption efficiency. While a denser lattice provides more contact nodes, progressively enlarging the unit cell and wire diameter yields a net positive effect on load capacity. For instance, HWS-S3 achieves TEA of 67.44 J and TSEA of 15,468.65 J/kg, which are 195.40% and 79.52% higher than those of the baseline HWS, respectively.
2. The deformation behavior of HWSs exhibits strong topological anisotropy. While weakening boundary constraints enhance out-of-plane (X-axis) energy absorption by promoting membrane flexibility and material draw-in, in-plane performance strictly relies on robust boundaries. Longitudinal (Y-axis) loading demands maximum boundary constraints to prevent unravelling, whereas transverse (Z-axis) loading exhibits an intermediate optimum where slight constraint relaxation aids loop reorientation and stiffening.
3. Specific HWS configurations exhibit distinct performance advantages tailored for different demands. Among the boundary-constrained models, HWS-X3 achieves the highest energy absorption (TEA: 40.31 J; TSEA: 15,212.20 J/kg), despite a slight reduction in PTF. Overall, the highest peak load-bearing capacity and absolute energy absorption are achieved by HWS-S3 (PTF: 5280.42 N; TEA: 67.44 J).

In summary, the HWSs proposed in this study possess substantial tensile toughness and stable deformation characteristics, enabling them to effectively absorb energy under complex loading conditions. This study systematically investigates the effects of various structural parameters on the tensile properties of the material, aiming to clarify the relationship between structural configuration and mechanical performance. These findings provide robust theoretical support and numerical analysis tools for the parametric design of such structures and open up new development directions for flexible protective components and adaptive structural designs. Crucially, several limitations remain that will guide our future research. First, while this study establishes a comprehensive numerical framework based on validated component-level material properties, the simulated results for the full macroscopic HWS configurations—which involve complex multi-body contact, friction, and large deformations—strictly remain numerical predictions. We are currently manufacturing full-scale physical prototypes of the HWS mesh; however, due to the complicated manufacturing processes required—such as ensuring consistent initial strand tension, controlling complex inter-wire friction, and designing custom clamping fixtures—the experimental validation will be comprehensively presented in our next phase of work. This upcoming research will definitively validate the reliability of HWSs before their practical deployment in complex engineering systems such as emergency escape ramps. Second, because the current evaluations rely on quasi-static loading, extrapolating these findings to impact-resistant applications requires further investigation into material strain rate dependencies, stress wave propagation, and dynamic inertial effects. Finally, the reported TEA and TSEA values have not yet been directly benchmarked against established commercial architectures under identical mass and loading conditions. Future investigations will prioritize this comparative benchmarking to definitively quantify the relative efficiency of HWSs within the broader landscape of flexible protective systems.

## Figures and Tables

**Figure 1:**
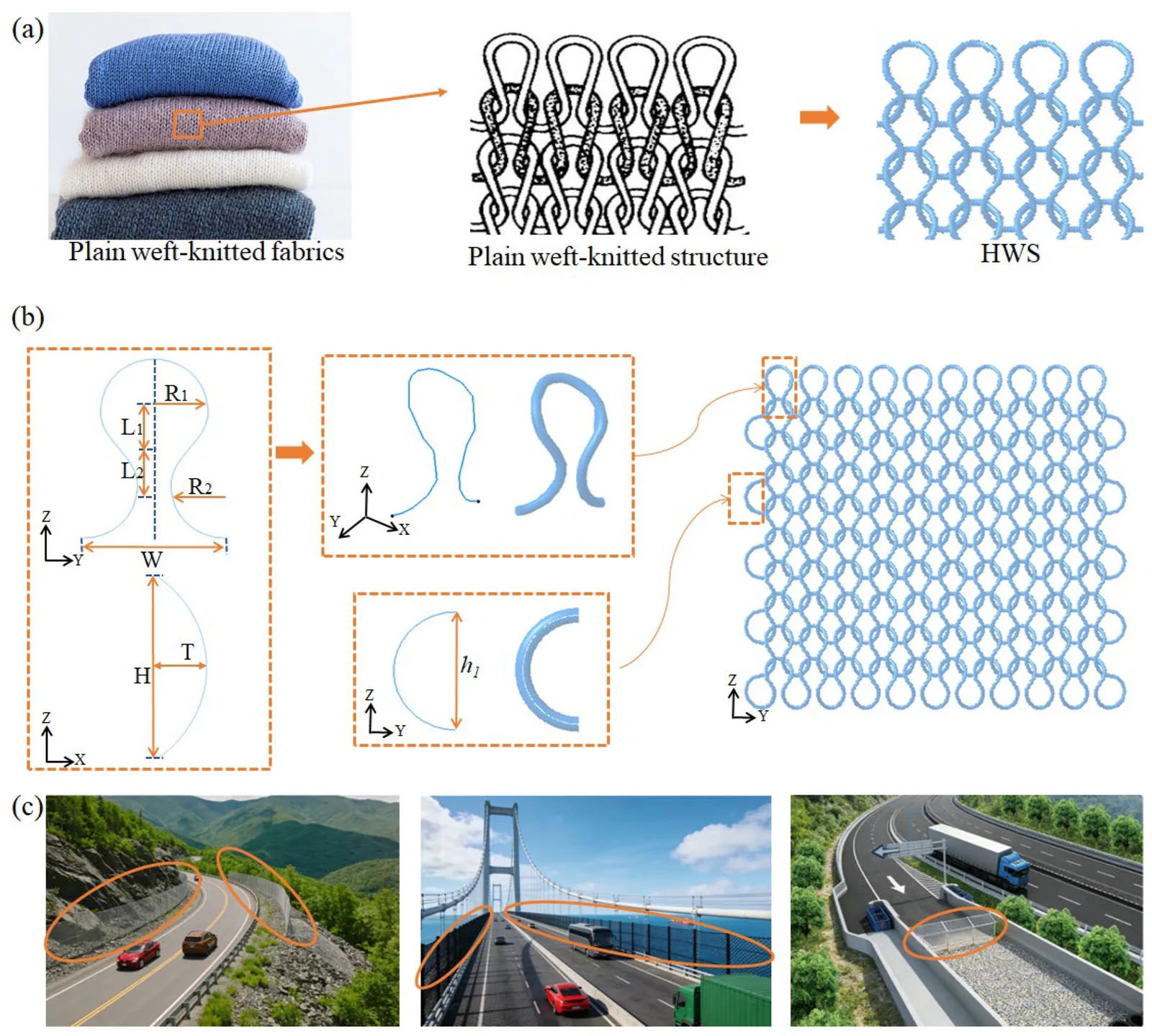
Specific design inspiration and application scenarios of HWSs. (**a**) The HWS inspired by plain weft-knitted structures. (**b**) Sketches are drawn on the YZ and XZ planes to generate unit curves and their corresponding unit structures, and then establish the HWS via arc line connection. (**c**) The potential application scenario of HWSs.

**Figure 2:**
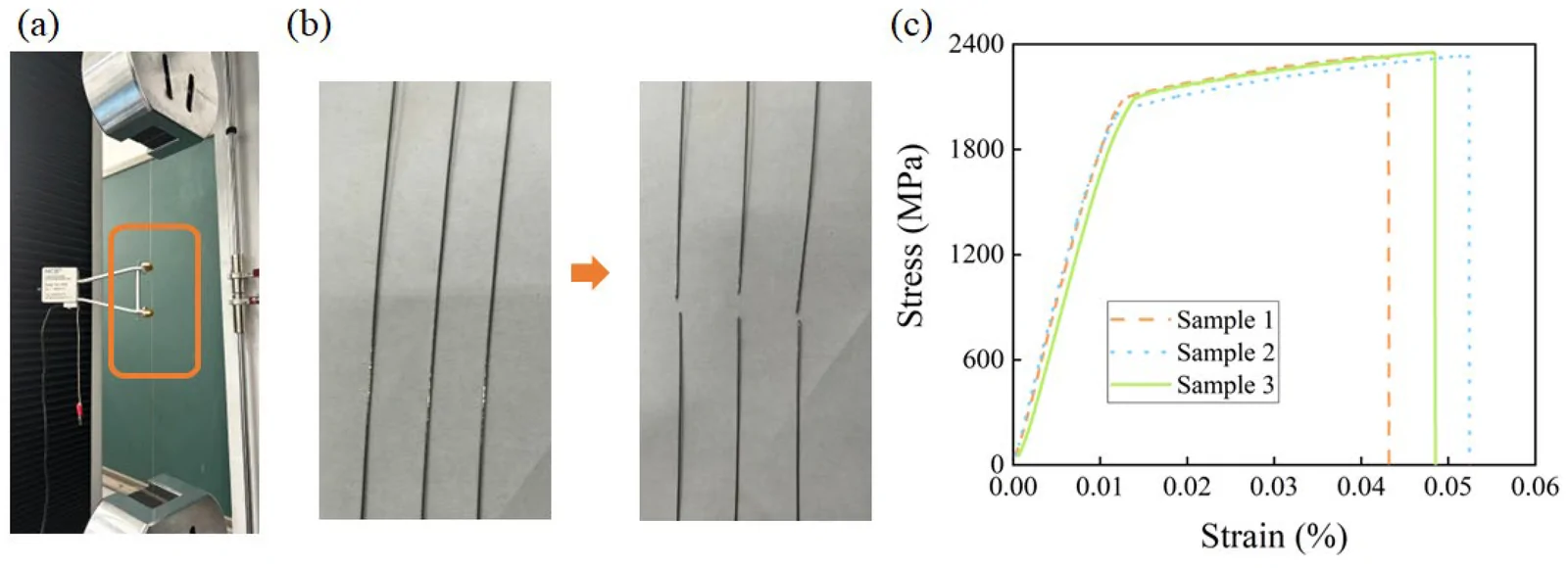
The tensile specimens and tensile tests. (**a**) The universal testing machine CMT-5105GL and installation of tensile sample. The upper end of the sample is completely fixed and constrained, while the lower end retains only the degree of freedom for vertical translational motion, applying a constant speed to stretch the steel wire rope. (**b**) The specimens before and after tensile tests. (**c**) The stress–strain curves obtained by tensile tests.

**Figure 3:**
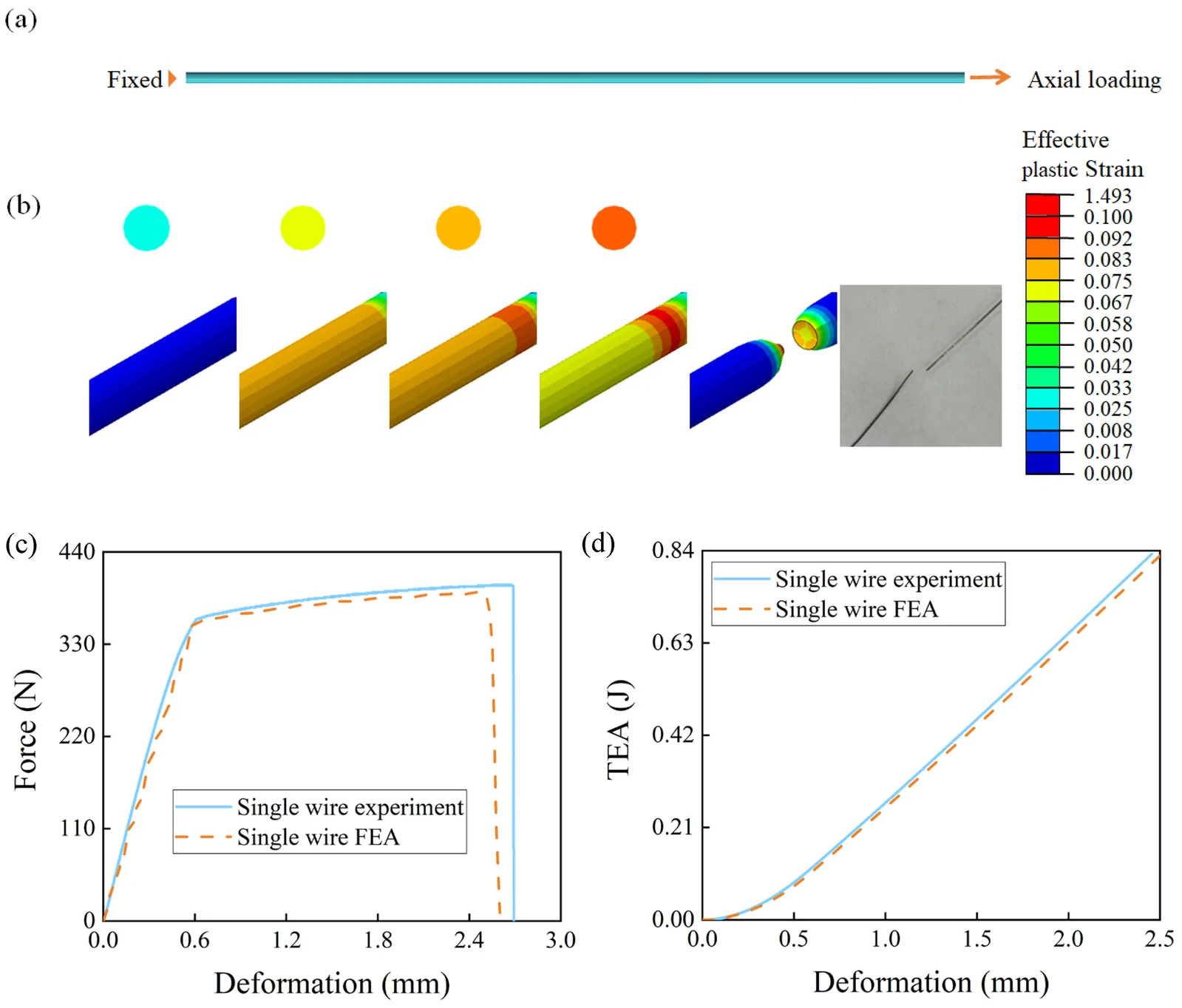
The comparison between experimental and FEA results of a single wire. The (**a**) finite element model and (**b**) strain cloud diagrams of a single wire. The (**c**) force–deformation and (**d**) TEA–deformation curves of a single wire. The single wire exhibits uniform tensile failure deformation in its cross-section, indicating homogeneous deformation characteristics and stable energy absorption performance.

**Figure 4:**
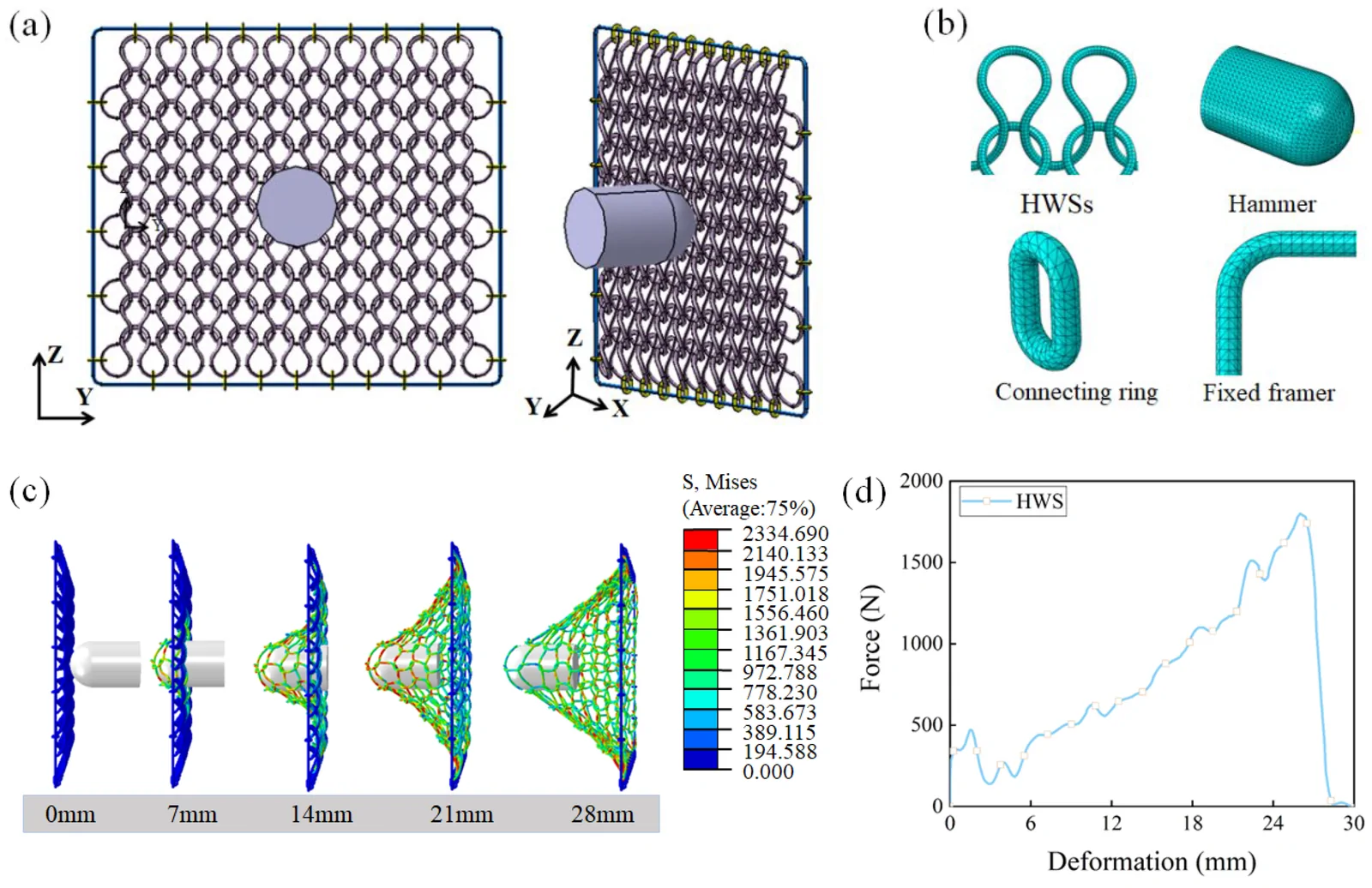
The FEA model and results of the HWS. (**a**) The FEA model and loading method of the HWS. (**b**) The meshing of the steel wire mesh, hammer, connecting ring, and fixed frame. (**c**) The stress contour plot of the HWS. (**d**) The force–deformation curve of the HWS.

**Figure 5:**
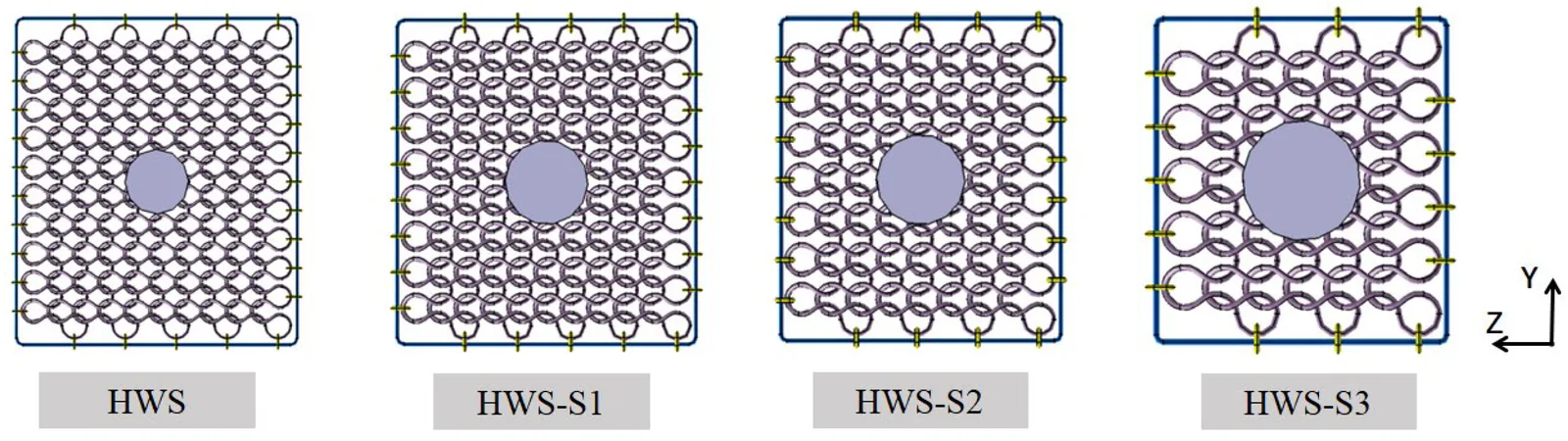
The front views of HWS, HWS-S1, HWS-S2 and HWS-S3.

**Figure 6:**
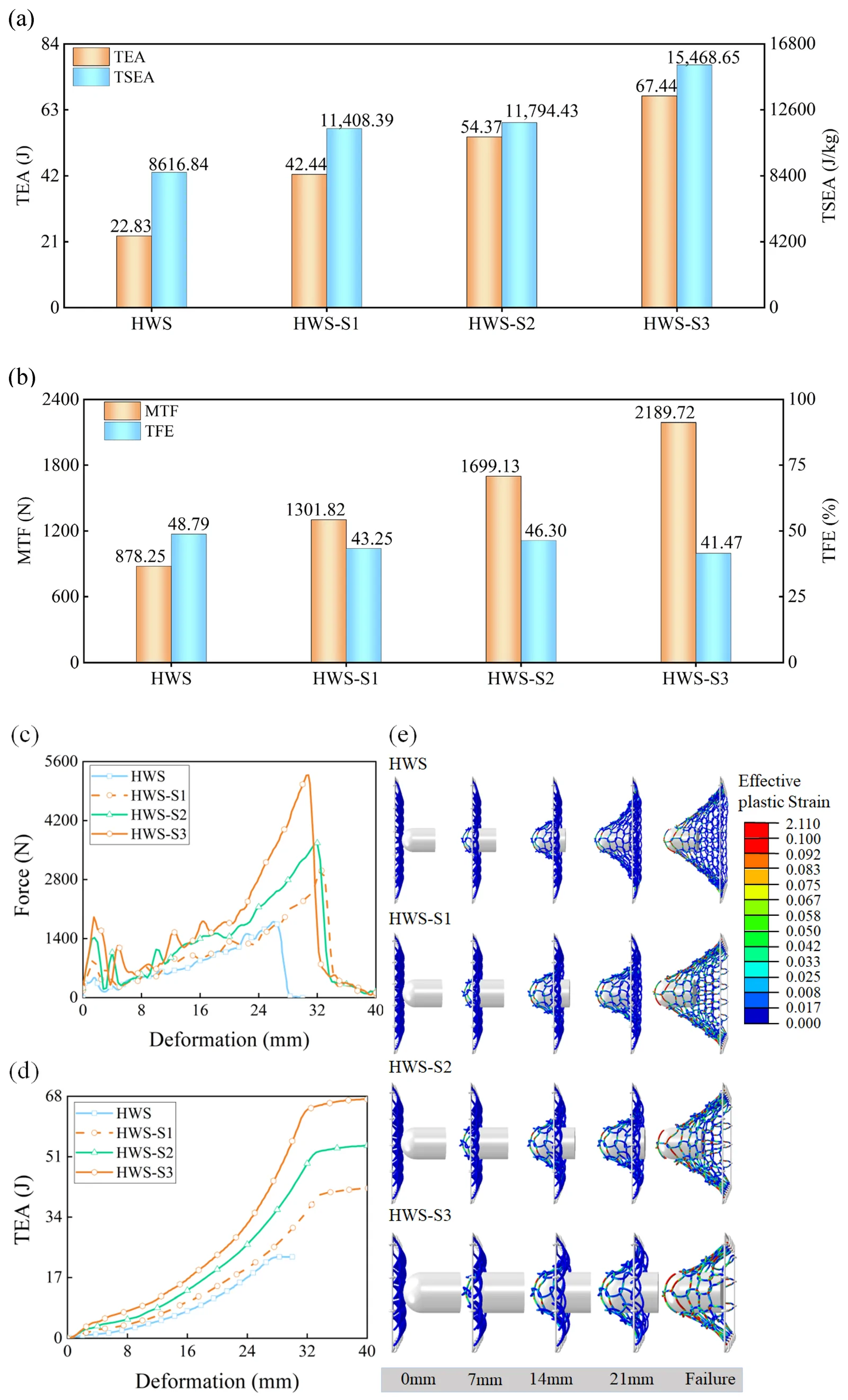

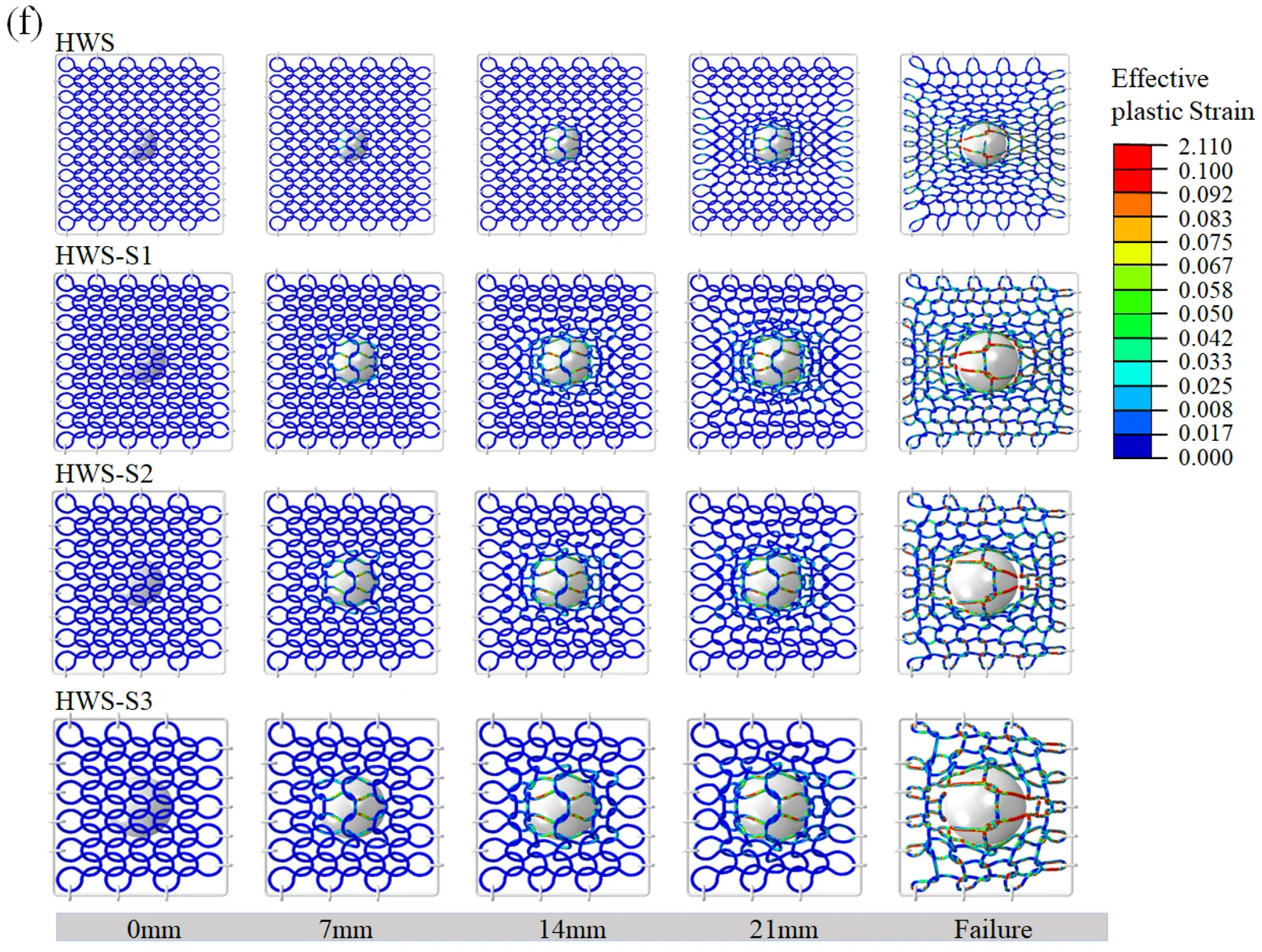
The analysis results of HWS, HWS-S1, HWS-S2 and HWS-S3. Histograms of (**a**) TEA-STEA and (**b**) MTF-TFE of these HWSs. The (**c**) force– and (**d**) TEA–deformation curves. (**e**) The right-side view of strain contour plots and (**f**) front view of strain contour plots for these HWSs.

**Figure 7:**
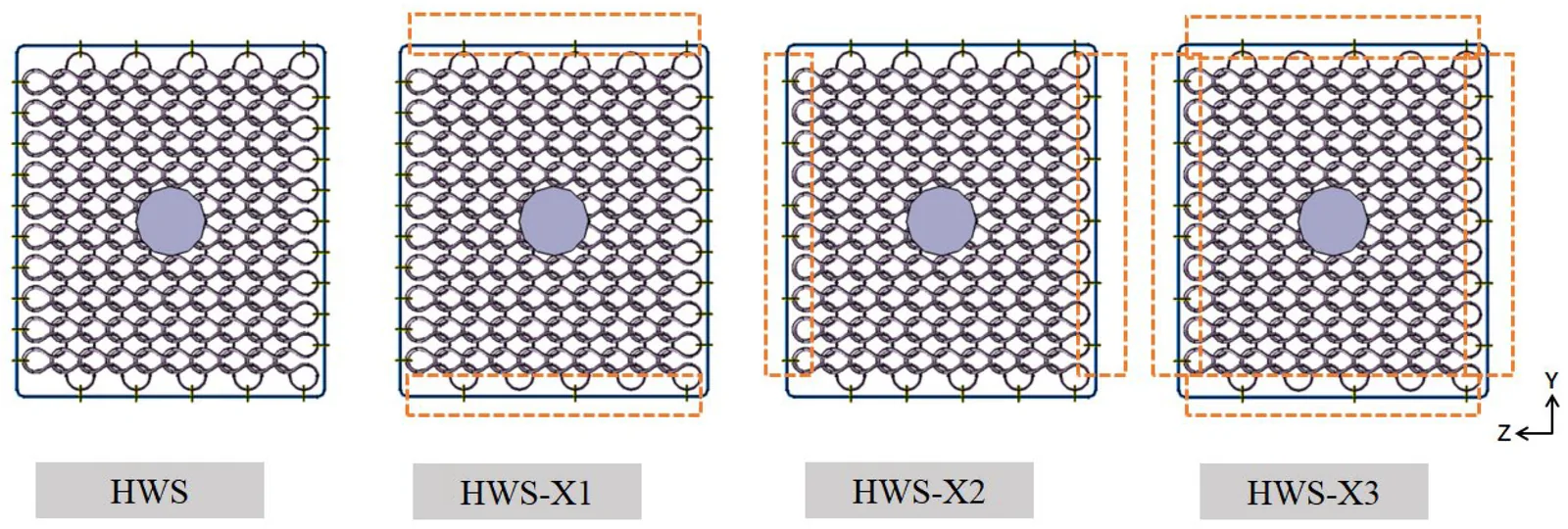
The front views of HWS, HWS-X1, HWS-X2 and HWS-X3.

**Figure 8:**
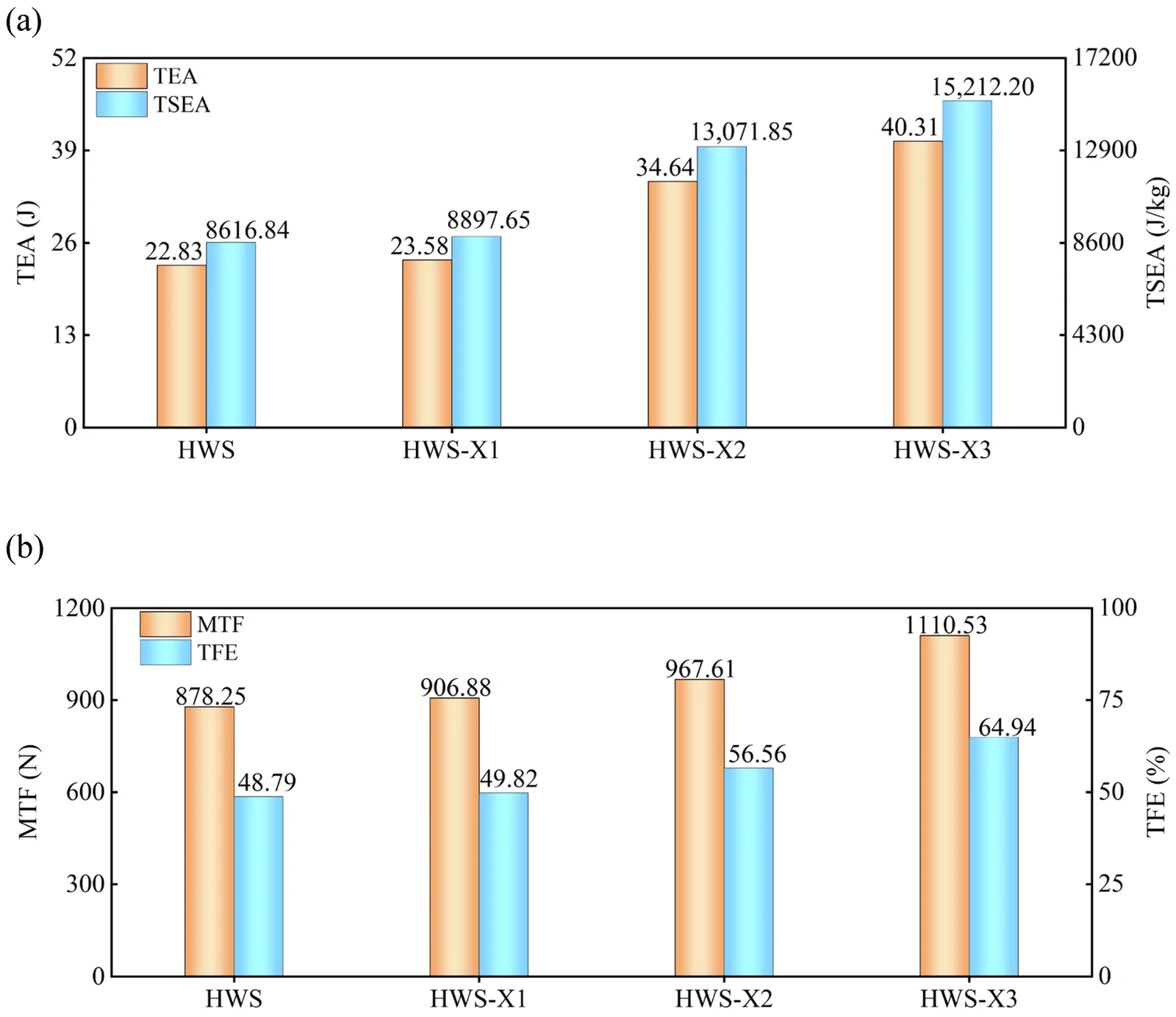

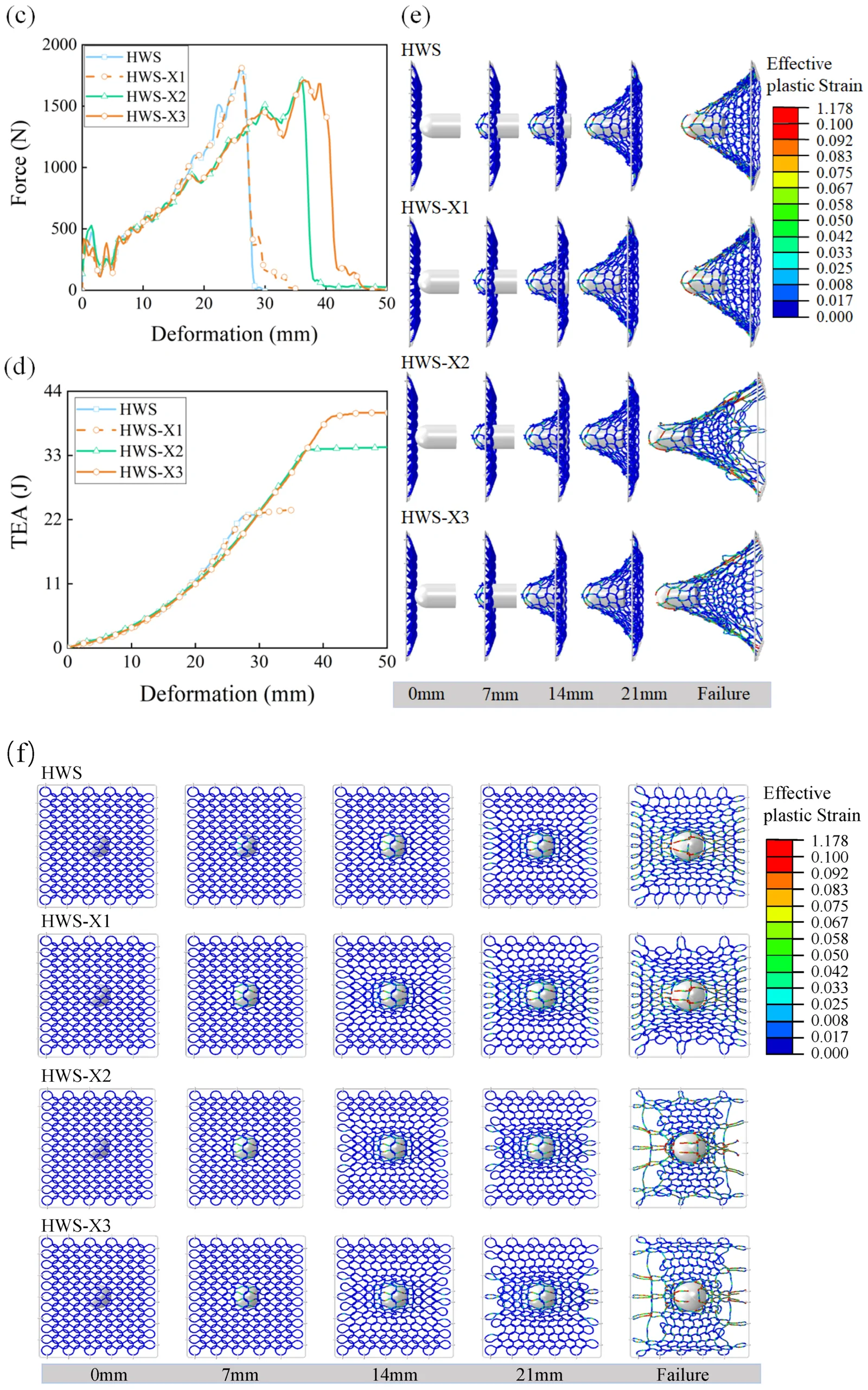
The analysis results of HWS, HWS-X1, HWS-X2 and HWS-X3. Histograms of (**a**) TEA-STEA and (**b**) MTF-TFE of these HWSs. The (**c**) force– and (**d**) TEA–deformation curves. (**e**) The right-side view of strain contour plots and (**f**) the front view of strain contour plots for these HWSs.

**Figure 9:**
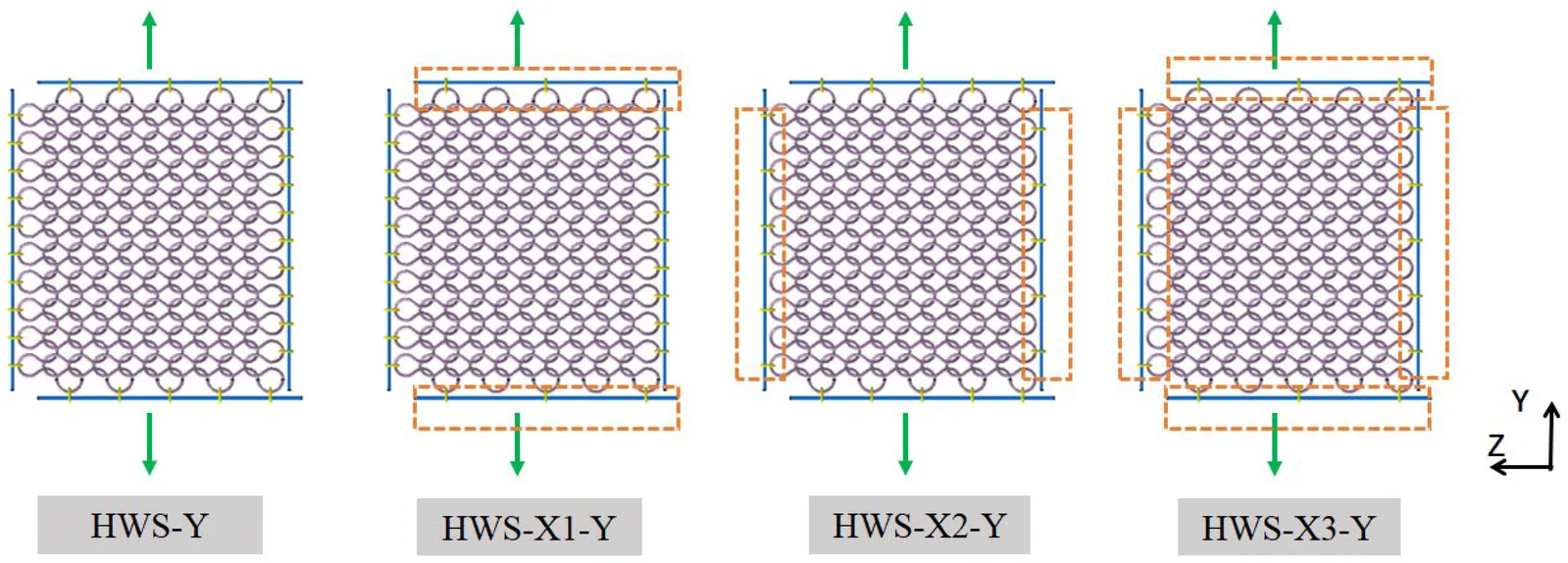
The front views of HWS-Y, HWS-X1-Y, HWS-X2-Y and HWS-X3-Y.

**Figure 10:**
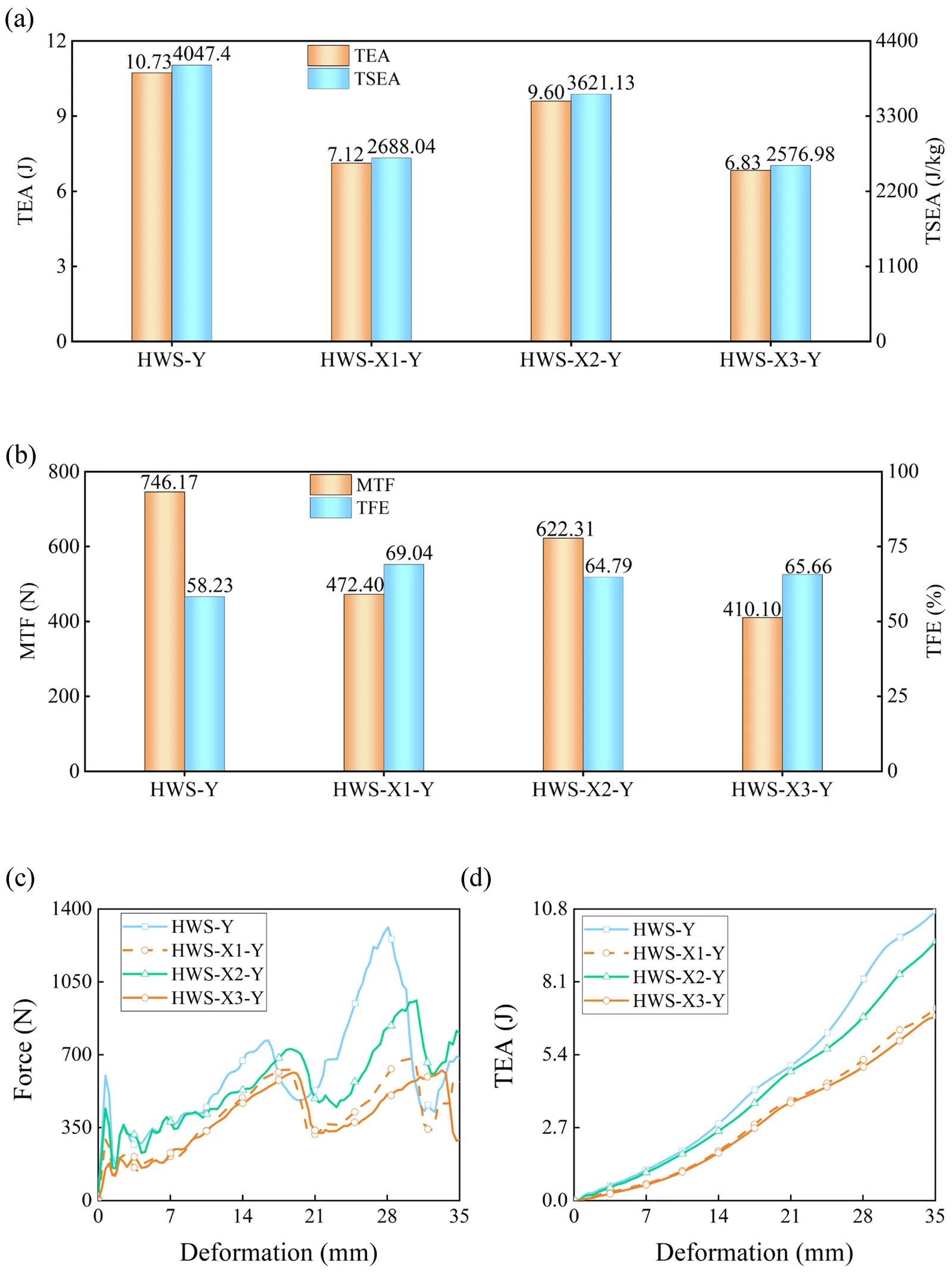

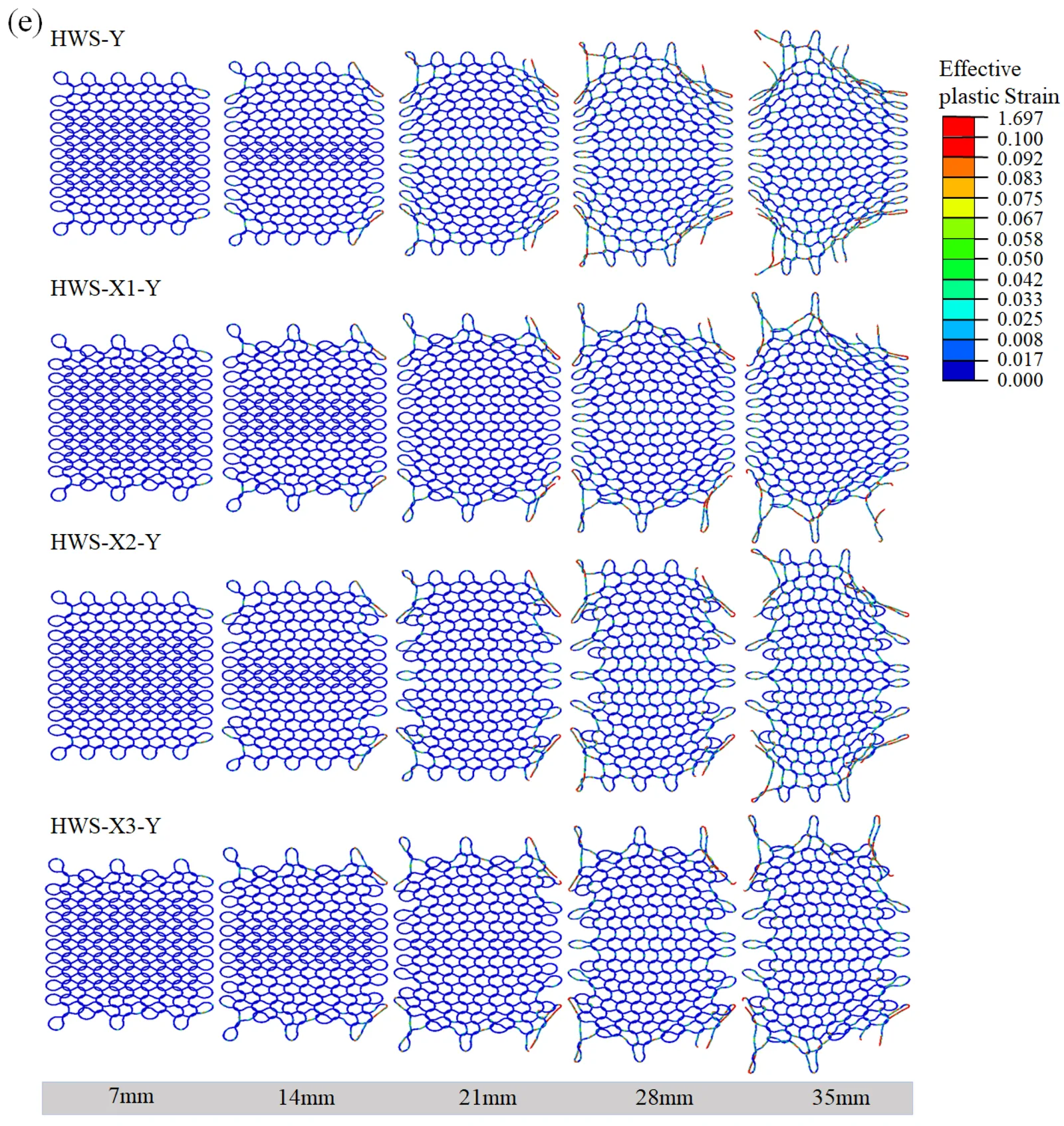
The analysis results of HWS-Y, HWS-X1-Y, HWS-X2-Y and HWS-X3-Y. Histograms of (**a**) TEA-STEA and (**b**) MTF-TFE of these HWSs. The (**c**) force– and (**d**) TEA–deformation curves. (**e**) The front view of strain contour plots for these HWSs.

**Figure 11:**
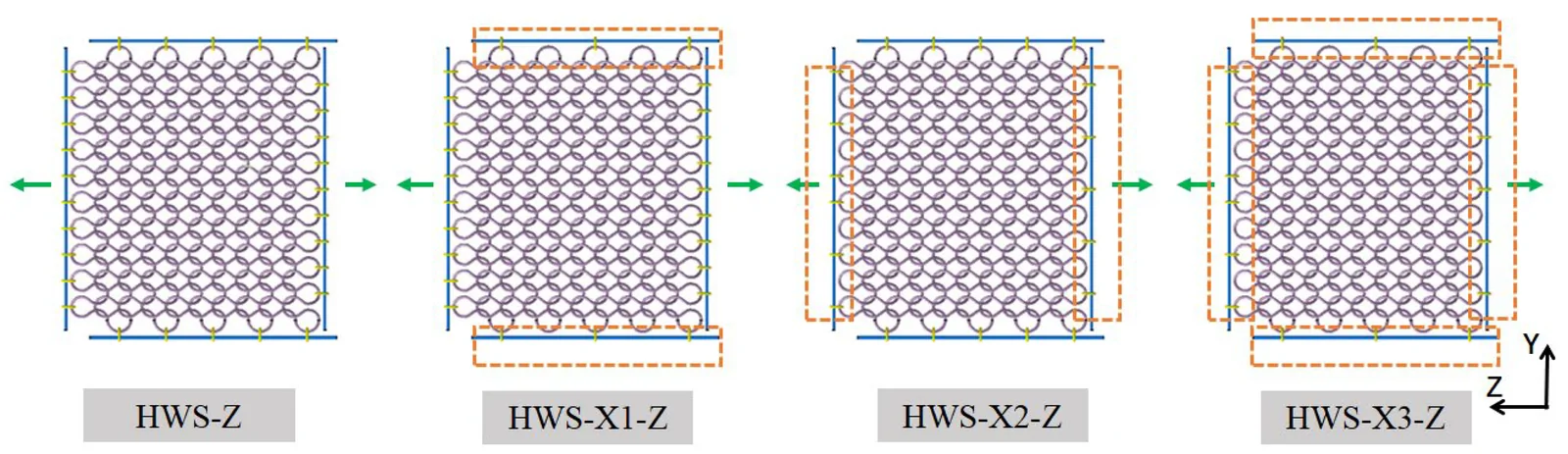
The front views of HWS-Z, HWS-X1-Z, HWS-X2-Z and HWS-X3-Z.

**Figure 12:**
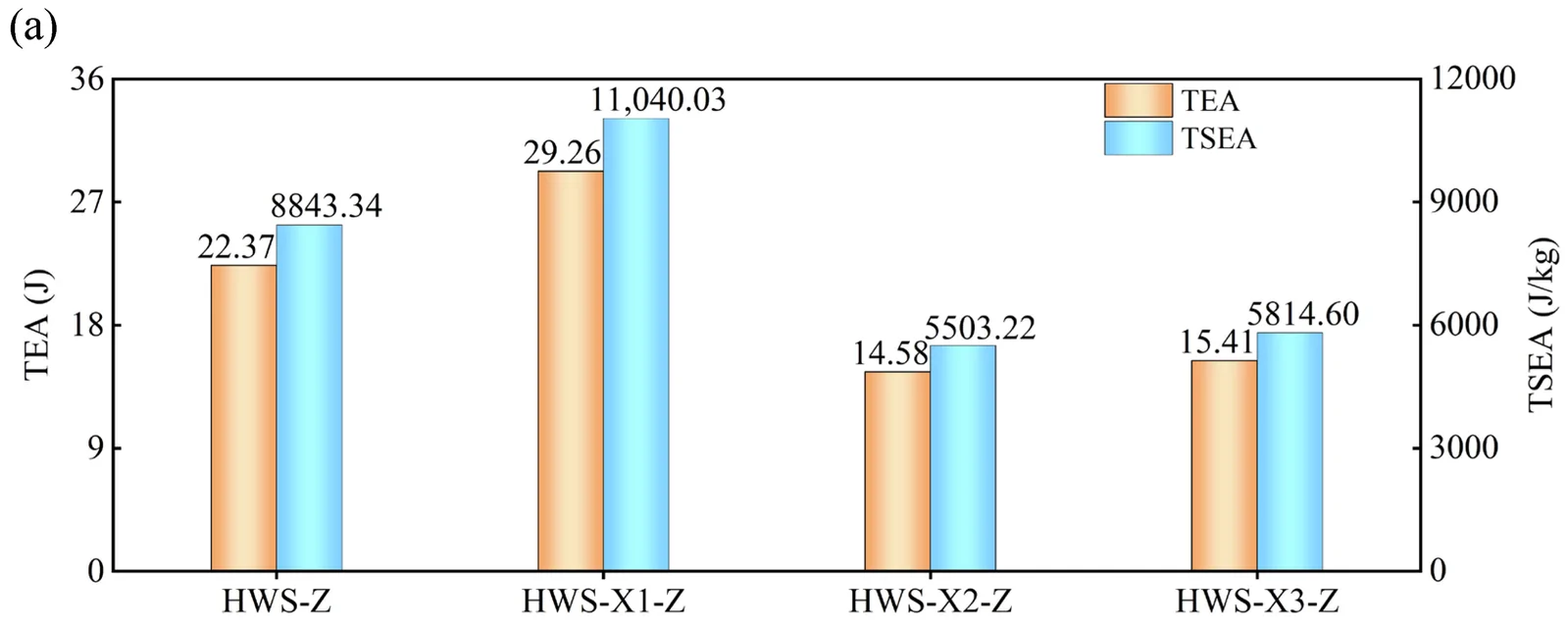

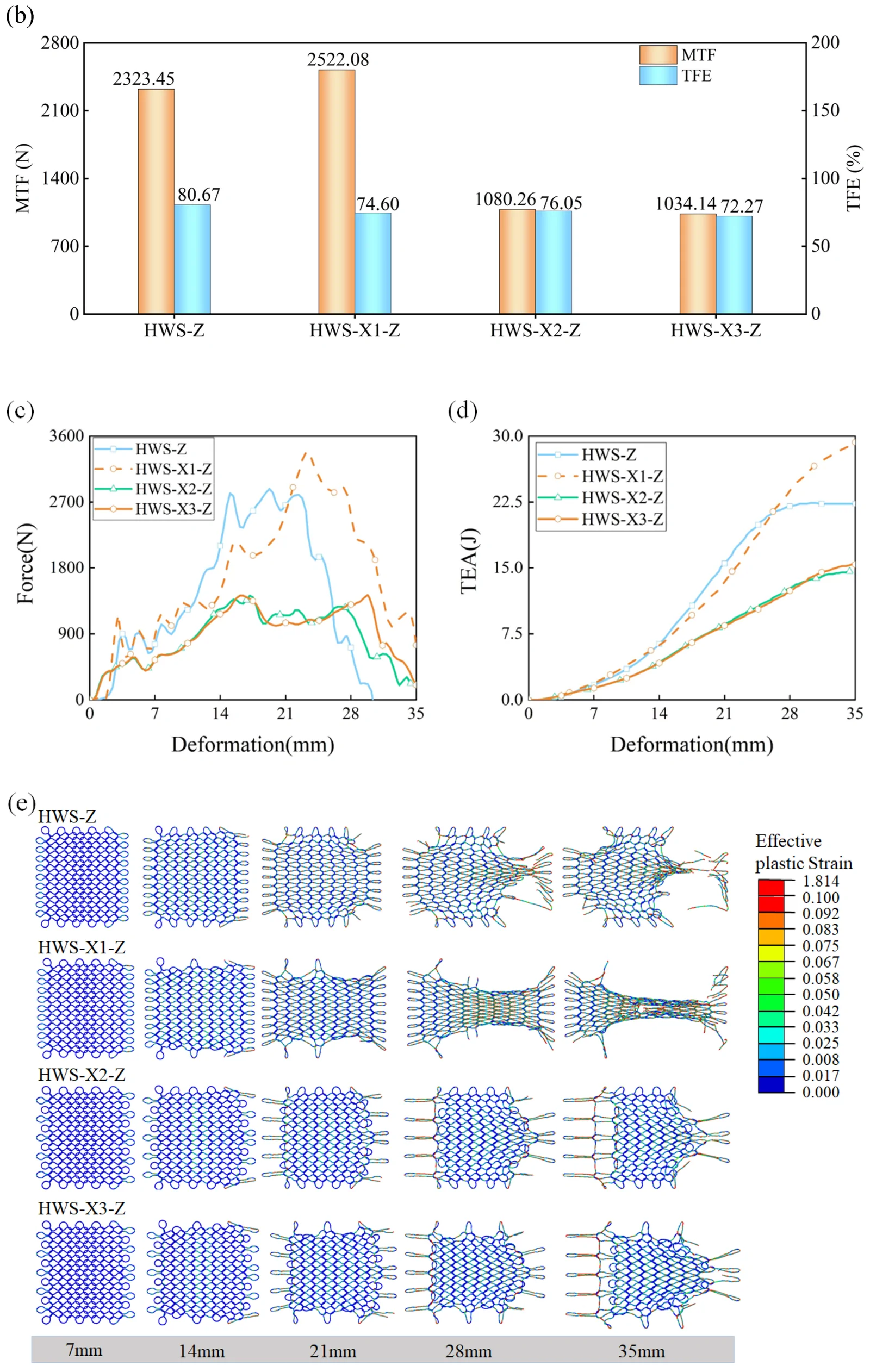
The analysis results of HWS-Z, HWS-X1-Z, HWS-X2-Z and HWS-X3-Z. Histograms of (**a**) TEA-STEA and (**b**) MTF-TFE of these HWSs. The (**c**) force– and (**d**) TEA–deformation curves. (**e**) The front view of strain contour plots for these HWSs.

**Table 1:** The structural parameters of the basic HWS model.

	L1 (mm)	L2 (mm)	R1 (mm)	R2 (mm)	W (mm)	H (mm)	T (mm)	h1 (mm)	Z	Y	d (mm)
HWS	1.38	1.38	1.67	1.67	4.44	6.1	1.5	4	10	10	0.48

**Table 2:** The material parameters of steel wire rope.

	**Ductile Damage**			
Density (kg/m^3^)	Fracture Strain	Stress Triaxiality	Strain Rate	Damage Evolution (mm)
7.85 × 10^3^	0.85	0.333	0	0.1
**Elastic**		**Plastic (Johnson–Cook)**		
Young’s modulus (MPa)	Poisson’s Ratio	A (MPa)	B (MPa)	n
174,968	0.3	2012.85	2774.65	0.65667

**Table 3:** Numerical values of single-wire tensile experiment and FEA.

		SM (g)	TEA (J)	TSEA (J/kg)	MTF (N)	PTF (N)	TFE
Single wire	FEA	0.0710	0.8289	11,674.23	334.76	391.88	85.4%
	Experiment	0.0710	0.8327	11,728.17	309.67	401.00	77.2%
	Error	0.00%	−0.46%	−0.46%	8.10%	−2.27%	10.62%

Note: “Error” is the difference between simulation and experimental results, and its expression is “Error = (FEA − experiment)/experiment”.

**Table 4:** The simulation results of the HWSs.

	SM (g)	TEA (J)	TSEA (J/kg)	MTF (N)	PTF (N)	TFE
HWSs	2.65	22.83	8616.84	878.25	1800.07	48.79%

**Table 5:** The detailed dimensions of the HWS-S models.

	L1 (mm)	L2 (mm)	R1 (mm)	R2 (mm)	W (mm)	H (mm)	T (mm)	h1 (mm)	Z	Y	d (mm)
HWS	1.38	1.38	1.67	1.67	4.44	6.1	1.5	4	10	10	0.48
HWS-S1	1.656	1.656	2.004	2.004	5.328	7.32	1.8	4	10	8	0.58
HWS-S2	2.001	2.001	2.422	2.422	6.438	8.864	2.175	5	8	7	0.70
HWS-S3	2.415	2.415	2.932	2.932	7.77	10.676	2.625	6	6	5	0.84

**Table 6:** The FEA results of the HWS-S models.

	SM (g)	TEA (J)	TSEA (J/kg)	MTF (N)	PTF (N)	TFE
HWS	2.65	22.83	8616.84	878.25	1800.07	48.79%
HWS-S1	3.72	42.44	11,408.39	1301.82	3010.03	43.25%
HWS-S2	4.61	54.37	11,794.43	1699.13	3670.15	46.30%
HWS-S3	4.36	67.44	15,468.65	2189.72	5280.42	41.47%

**Table 7:** The FEA results of the HWS-X models.

	SM (g)	TEA (J)	TSEA (J/kg)	MTF (N)	PTF (N)	TFE
HWS	2.65	22.83	8616.84	878.25	1800.07	48.79%
HWS-X1	2.65	23.58	8897.65	906.88	1820.44	49.82%
HWS-X2	2.65	34.64	13,071.85	967.61	1710.68	56.56%
HWS-X3	2.65	40.31	15,212.20	1110.53	1710.15	64.94%

**Table 8:** The FEA results of the HWS-Y models.

	SM (g)	TEA (J)	TSEA (J/kg)	MTF (N)	PTF (N)	TFE
HWS-Y	2.65	10.73	4047.40	746.17	1312.37	58.23%
HWS-X1-Y	2.65	7.12	2688.04	472.40	680.68	69.04%
HWS-X2-Y	2.65	9.60	3621.13	622.31	960.49	64.79%
HWS-X3-Y	2.65	6.83	2576.98	410.10	624.58	65.66%

**Table 9:** The FEA results of the HWS-Z models.

	SM (g)	TEA (J)	TSEA (J/kg)	MTF (N)	PTF (N)	TFE
HWS-Z	2.65	22.37	8443.34	2323.45	2880.17	80.67%
HWS-X1-Z	2.65	29.26	11,040.03	2522.08	3380.69	74.60%
HWS-X2-Z	2.65	14.58	5503.22	1080.26	1420.44	76.05%
HWS-X3-Z	2.65	15.41	5814.60	1034.14	1430.99	72.27%

## Data Availability

The raw data supporting the conclusions of this article will be made available by the authors on request.

## Supplementary Materials

The following supporting information can be downloaded at: https://www.mdpi.com/article/10.3390/s26196080/s1, Figure S1: Parametric sensitivity analysis evaluating the effect of mesh element size on the finite element predictions; Figure S2: Parametric sensitivity analysis evaluating the effect of critical damage displacement on the finite element predictions.

## Abbreviations

The following abbreviations are used in this manuscript:

HWSs	heuristic weaving structures
FEA	finite element analysis
TEA	tensile energy absorption
TSEA	tensile specific energy absorption
PTF	peak tensile force
MTF	mean tensile force
TFE	tensile force efficiency
HWS	the baseline sparsity configuration features a dense lattice distribution with characteristic dimensions of W = 4.44 mm, H = 6.1 mm, and lattice counts of Z = 10 and Y = 10
HWS-S1	with its core layout unchanged, the first sparse configuration increases characteristic geometric dimensions to W = 5.328 mm and H = 7.32 mm while adjusting the lattice distribution to Z = 10 and Y = 8
HWS-S2	the second sparse configuration further expands the contour and overall width to W = 6.438 mm and H = 8.864 mm while reducing the lattice counts to Z = 8 and Y = 7
HWS-S3	the sparsest configuration enlarges the geometric dimensions to W = 7.77 mm and H = 10.676 mm while lowering the sparse lattice distribution to Z = 6 and Y = 5
HWS-X1	connecting rings at the longitudinal ends are halved compared to the baseline, with the transverse ends remaining unchanged
HWS-X2	connecting rings at the transverse ends are halved compared to the baseline, with the longitudinal ends remaining unchanged
HWS-X3	connecting rings across all boundaries—both longitudinal and transverse ends—are halved compared to the baseline
HWS-Y	the baseline model under Y-axis tensile loading consists of 10 and 9 connecting rings at the left and right transverse ends, respectively, and 5 connecting rings at each of the upper and lower longitudinal ends
HWS-X1-Y	subjected to Y-axis tensile loading, the number of connecting rings at the upper and lower longitudinal ends is reduced to half of the baseline value, while the transverse ends remain unchanged
HWS-X2-Y	subjected to Y-axis tensile loading, the number of connecting rings at the left and right transverse ends is reduced to half of the baseline value, while the longitudinal ends remain unchanged
HWS-X3-Y	subjected to Y-axis tensile loading, the number of connecting rings at both the upper and lower longitudinal ends and the left and right transverse ends is reduced to half of the baseline value
HWS-Z	the baseline model under Z-axis tensile loading consists of 10 and 9 connecting rings at the left and right transverse ends, respectively, and 5 connecting rings at each of the upper and lower longitudinal ends
HWS-X1-Z	subjected to Z-axis tensile loading, the number of connecting rings at the upper and lower longitudinal ends is reduced to half of the baseline value, while the transverse ends remain unchanged
HWS-X2-Z	subjected to Z-axis tensile loading, the number of connecting rings at the left and right transverse ends is reduced to half of the baseline value, while the longitudinal ends remain unchanged
HWS-X3-Z	subjected to Z-axis tensile loading, the number of connecting rings at both the upper and lower longitudinal ends and the left and right transverse ends is reduced to half of the baseline value
