# Supplementary material for: Tensile Property and Parametric Study of Heuristic Weaving Structures
Source: Sensors (Basel). 2026 Sep 25;26(19):6080. doi: 10.3390/s26196080 (PMC13667665; doi:10.3390/s26196080)
Supplement: Supplementary file 1 [file sensors-26-06080-s001.zip › sensors-4518373-supplementary.pdf]

sensors

MDPI

Supplementary Material for Tensile Property and Parametric Study of Heuristic

Jing Xiao [1], Qiqi Li [2,*], Fang Wang [2], Tiefang Zou [2] and Kai Gao [2] and Lin Hu [2]

[1] School of Transportation, Changsha University of Science & Technology, Changsha 410114, China
[2] College of Mechanical and Vehicle Engineering, Changsha University of Science & Technology, Changsha 410114, China
* Correspondence: liqiqi@csust.edu.cn

1. The Sensitivity Analysis of Mesh

To evaluate the influence of the mesh element size on the mechanical response of the steel wire rope, a sensitivity analysis was performed by considering different global mesh sizes in the finite element model. Four values, namely 0.05 mm, 0.1 mm, 0.15 mm, and 0.2 mm, were investigated while keeping all other material parameters and modeling conditions unchanged.

The predicted force–deformation responses for different mesh sizes are compared with the experimental result in **Figure S1**(e). The results indicate that the mesh size has a noticeable influence on the load-bearing capacity and failure stage of the wire rope. A smaller value (0.05 mm) leads to an overestimation of the peak tensile force. In contrast, larger values (0.15 mm and 0.2 mm) result in an underestimation of the structural stiffness and exhibit premature failure behavior during the deformation process.

Among the investigated cases, the simulation with a mesh size of 0.1 mm shows the closest agreement with the experimental force–deformation curve. The predicted peak tensile force and failure deformation are consistent with the experimental observations, indicating that this value provides a reasonable description of the structural deformation and fracture evolution process under the current modeling conditions.

Furthermore, the influence of the mesh size on the tensile energy absorption (TEA) was evaluated, as shown in **Figure S1**(f). The TEA obtained using 0.1 mm agrees well with the experimental trend, while deviations increase when the mesh size is reduced to 0.05 mm or increased to 0.15 mm and 0.2 mm.

Based on the above sensitivity analysis, the mesh size of 0.1 mm was selected for subsequent simulations. It should be noted that this parameter governs the balance between computational efficiency and numerical accuracy. Although the sensitivity analysis demonstrates that 0.1 mm provides a reliable prediction under the current modeling conditions, further investigation using significantly finer meshes could drastically increase computational costs without yielding a proportional increase in calibration accuracy.

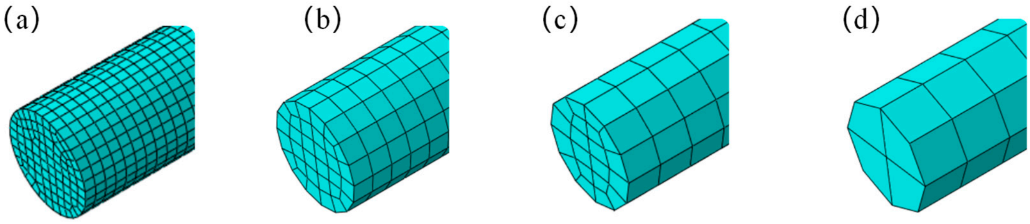

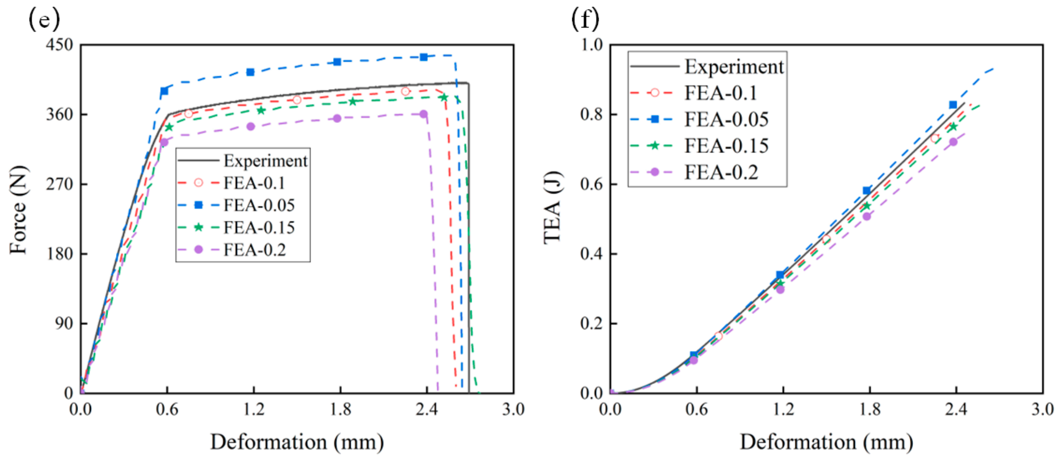

Figure S1. Parametric sensitivity analysis evaluating the effect of mesh element size on the finite element predictions. (a)–(d) The finite element mesh configurations of the single steel wire with global element sizes of 0.05 mm, 0.1 mm, 0.15 mm, and 0.2 mm, respectively. (e) Comparison of Force-Deformation curves between the experimental data and FEA models utilizing different mesh sizes, highlighting force overestimation at 0.05 mm and premature failure at coarser mesh values. (f) Comparison of Tensile Energy Absorption (TEA)-Deformation curves, demonstrating the calibration accuracy and optimal convergence of the 0.1 mm configuration.

2. The Sensitivity Analysis of Damage Parameters

To evaluate the influence of the damage evolution displacement on the mechanical response of the steel wire rope, a sensitivity analysis was performed by considering different damage evolution displacement values in the Abaqus ductile damage model. Four values, namely 0.05 mm, 0.10 mm, 0.15 mm, and 0.20 mm, were investigated while keeping all other material parameters and modeling conditions unchanged.

The predicted force–deformation responses for different damage evolution displacement values are compared with the experimental result in **Figure S2**(a). The results indicate that the damage evolution displacement has a noticeable influence on the post-yield behavior and failure stage of the wire rope. A smaller value (0.05 mm) leads to earlier stiffness degradation and a relatively delayed representation of the deformation process after damage initiation. In contrast, larger values (0.15 mm and 0.20 mm) result in a slower damage evolution and underestimate the degradation behavior during the failure stage.

Among the investigated cases, the simulation with a damage evolution displacement of 0.10 mm shows the closest agreement with the experimental force–deformation curve. The predicted peak tensile force and failure deformation are consistent with the experimental observations, indicating that this value provides a reasonable description of the fracture evolution process under the current modeling conditions.

Furthermore, the influence of the damage evolution displacement on the tensile energy absorption (TEA) was evaluated, as shown in **Figure S2**(b). The TEA obtained using 0.10 mm agrees well with the experimental trend, while deviations increase when the damage evolution displacement is reduced to 0.05 mm or increased to 0.15 mm and 0.20 mm.

Based on the above sensitivity analysis, the damage evolution displacement of 0.10 mm was selected for subsequent simulations. It should be noted that this parameter is associated with the adopted finite element discretization and damage evolution formulation. Although the sensitivity analysis demonstrates that 0.10 mm provides reliable prediction under the current modeling conditions, further investigation considering different mesh configurations may provide additional insight into the mesh dependency of the damage evolution behavior.

https://doi.org/10.3390/s26196080

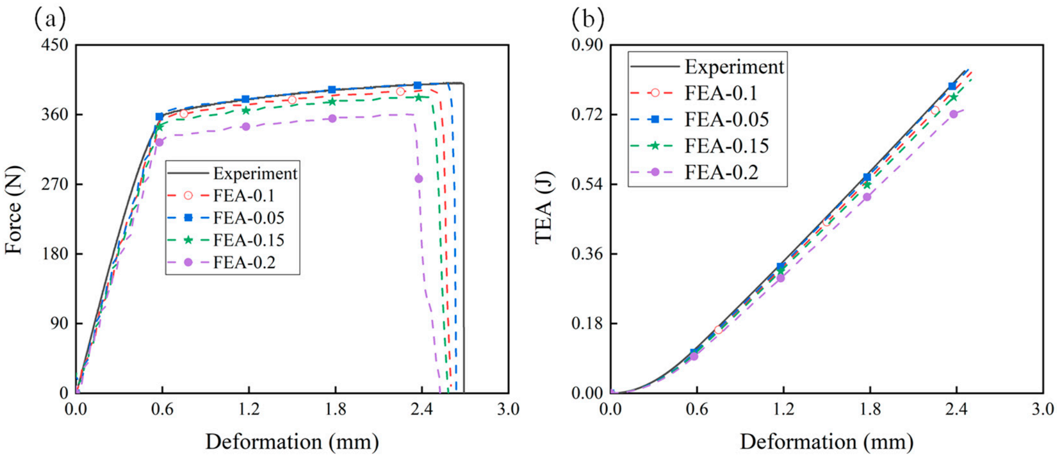

Figure S2. Parametric sensitivity analysis evaluating the effect of critical damage displacement on the finite element predictions. (a) Comparison of Force-Deformation curves between the experimental data and FEA models utilizing critical displacements of 0.05 mm, 0.10 mm, 0.15 mm, and 0.20 mm, lower values lead to premature failure. (b) Comparison of Tensile Energy Absorption (TEA)-Deformation curves, demonstrating the calibration accuracy and optimal convergence of the 0.10 mm configuration.

Disclaimer/Publisher's Note: The statements, opinions and data contained in all publications are solely those of the individual author(s) and contributor(s) and not of MDPI and/or the editor(s). MDPI and/or the editor(s) disclaim responsibility for any injury to people or property resulting from any ideas, methods, instructions or products referred to in the content.

https://doi.org/10.3390/s26196080
